# Abl Kinases Regulate HGF/Met Signaling Required for Epithelial Cell Scattering, Tubulogenesis and Motility

**DOI:** 10.1371/journal.pone.0124960

**Published:** 2015-05-06

**Authors:** Ran Li, Jennifer F. Knight, Morag Park, Ann Marie Pendergast

**Affiliations:** 1 Department of Pharmacology and Cancer Biology, Duke University School of Medicine, Durham, North Carolina, United States of America; 2 Goodman Cancer Research Centre, McGill University, Montreal, QC, Canada; 3 Departments of Biochemistry and Oncology, McGill University, Montreal, QC, Canada; Thomas Jefferson University, UNITED STATES

## Abstract

Tight regulation of receptor tyrosine kinases (RTKs) is crucial for normal development and homeostasis. Dysregulation of RTKs signaling is associated with diverse pathological conditions including cancer. The Met RTK is the receptor for hepatocyte growth factor (HGF) and is dysregulated in numerous human tumors. Here we show that Abl family of non-receptor tyrosine kinases, comprised of Abl (*ABL1*) and Arg (*ABL2*), are activated downstream of the Met receptor, and that inhibition of Abl kinases dramatically suppresses HGF-induced cell scattering and tubulogenesis. We uncover a critical role for Abl kinases in the regulation of HGF/Met-dependent RhoA activation and RhoA-mediated actomyosin contractility and actin cytoskeleton remodeling in epithelial cells. Moreover, treatment of breast cancer cells with Abl inhibitors markedly decreases Met-driven cell migration and invasion. Notably, expression of a transforming mutant of the Met receptor in the mouse mammary epithelium results in hyper-activation of both Abl and Arg kinases. Together these data demonstrate that Abl kinases link Met activation to Rho signaling and Abl kinases are required for Met-dependent cell scattering, tubulogenesis, migration, and invasion. Thus, inhibition of Abl kinases might be exploited for the treatment of cancers driven by hyperactivation of HGF/Met signaling.

## Introduction

Receptor tyrosine kinases (RTKs) regulate processes required for normal mammalian development and tissue homeostasis, and aberrant RTK function promotes numerous pathological conditions including cancer [1]. Among these RTKs, the hepatocyte growth factor (HGF) receptor, Met, regulates vertebrate cellular responses including proliferation, survival, motility, invasion and morphogenesis required for normal physiological functions during embryogenesis and in the adult [1, 2]. Enhanced HGF/Met signaling is a characteristic feature of many solid tumors and is associated with therapy resistance [1–4]. Up-regulation of HGF/Met signaling may occur through *MET* amplification, transcriptional activation, Met protein kinase activation, and/or increased HGF production [5, 6]. *MET* amplification or activating mutations have been detected in gastric and lung cancers [7–13], while transcriptional up-regulation has been reported in breast, colorectal and liver cancers [14–16]. Enhanced Met activity can promote cancer cell proliferation, survival, migration and/or invasion [17]. Recently, Met was shown to promote invadopodia, which are actin-rich, matrix-degrading membrane structures, in breast and gastric carcinoma cell lines [18]. A number of inhibitors targeting the HGF/Met pathway are currently under development for the treatment non-small-cell lung cancer (NSCLC) and other solid tumors [19]. Met activation results in the phosphorylation of tyrosines Y1234/1235 in the kinase domain and carboxy-terminal tyrosines Y1349/1356 for substrate docking and downstream signaling leading to diverse cellular responses [2, 20]. It is unclear whether the multiple cytoskeletal responses regulated by Met are coordinated by common or distinct signaling mediators.

The Abl non-receptor tyrosine kinases, Abl (*ABL1*) and Arg (*ABL2*), regulate cytoskeletal processes required for cell polarity, morphogenesis, epithelial-to-mesenchymal transition (EMT), migration, and invasion [21–26]. Abl kinases were first identified as oncogenes in leukemias, but recent reports have demonstrated a functional role for *ABL1* and/or *ABL2* in solid tumors including breast carcinoma, kidney cancer and melanoma [27–29]. Similar to the Met receptor, Abl kinases target several Rho family GTPases and actin regulatory proteins to induce morphogenetic events during normal development and cancer [21, 22, 30–33]. However, whether Abl kinases play a mechanistic role in the regulation of Met-dependent epithelial cell scattering and tubulogenesis is unclear. Here we report that Abl kinases link Met receptor activation to RhoA signaling leading to actomyosin contractility in epithelial cells, and demonstrate that inhibition of Abl kinases suppresses HGF-induced cytoskeletal remodeling processes required for cell scattering and tubulogenesis of non-transformed Madin Darby canine kidney (MDCK) epithelial cells, as well as migration and invasion of breast cancer cells.

## Results

### Abl Kinases Are Activated by HGF/Met and Promote HGF-induced Cell Scattering

**Fig 1:**
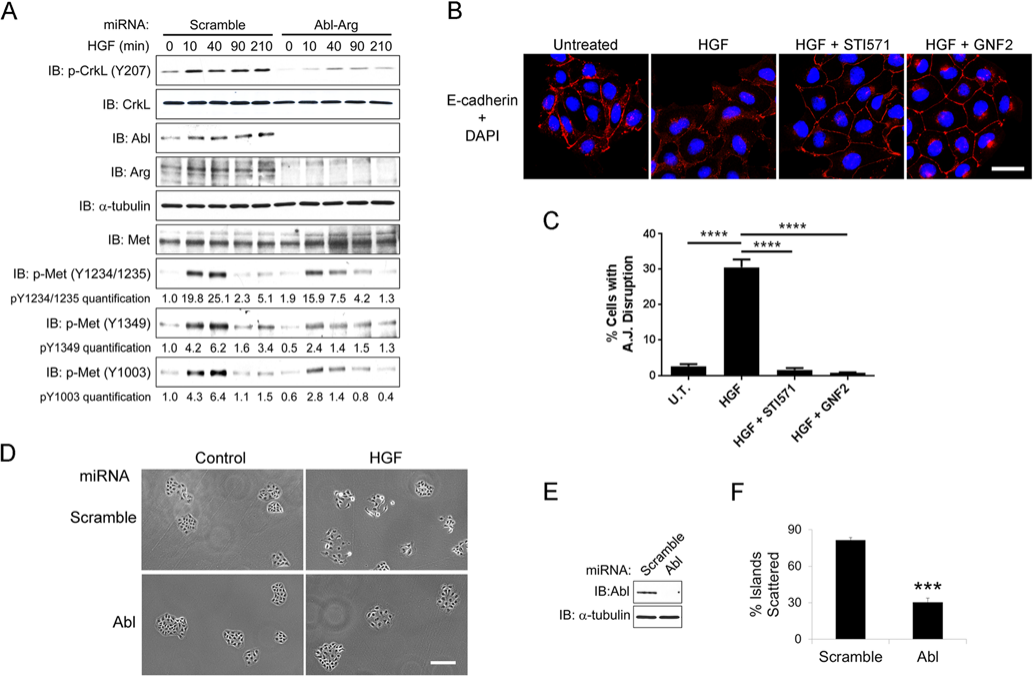
Abl kinases are activated by the Met receptor and promote HGF-induced cell scattering. (A) Abl kinases are activated downstream of the Met receptor. Serum-starved MDCK cells expressing either scramble control or Abl-Arg dual miRNAs were treated with 20ng/ml HGF for the indicated times. Cells were lysed and the lysates were subjected to western blotting with the indicated antibodies. Tubulin was used as loading control. Phosphorylation of the Met receptor on the indicated tyrosine residues was normalized to total Met protein and quantified by ImageJ. (B-C) Serum-starved MDCK cells were either left untreated or treated with HGF for 3 hours in the presence or absence of Abl kinases inhibitors STI571 (15μm) or GNF2 (20μm). Cells were fixed and stained for E-cadherin followed by fluorescence microscopy analysis (B). The percentages of cells with disrupted adherens junctions from each experimental group were quantified and analyzed by one-way ANOVA followed by Bonferroni post-test. ****P<0.0001. Error bars represent mean (n = 3) ± S.E.M. (C). (D-F) Loss of Abl kinase impairs HGF-induced cell scattering. MDCK cells expressing the indicated miRNAs were plated sparsely and allowed to form cell islands. Cells were serum-starved overnight and treated with HGF for 7 hours. Cells were fixed and analyzed by microscopy; bright field pictures are shown (D). Knockdown of the Abl kinase is confirmed by western blotting (E). The percentages of islands scattered were quantified and analyzed by two-tailed unpaired Student’s t-test. ***P<0.001. Error bars represent mean (n = 3) ± S.E.M. (F).

To evaluate the role of Abl kinases in Met-dependent cytoskeletal remodeling, we employed non-transformed Madin Darby canine kidney (MDCK) cell line, a widely-used model in studies of HGF-induced epithelial-mesenchymal transition (EMT) and cell spreading [34]. In response to HGF treatment, we observed increased phosphorylation of CrkL on tyrosine 207, an Abl- and Arg- specific phosphorylation site (Fig 1A). This phosphorylation was markedly decreased in cells depleted of both Abl and Arg kinases by miRNA-mediated knockdown (Fig 1A) supporting HGF/Met-induced activation of Abl family kinases in MDCK cells. As HGF is known to cause scattering of MDCK cells, we examined the role of Abl kinases in this process using two distinct pharmacological inhibitors of the Abl kinases: STI571 (Imatinib/Gleevec) and GNF2, which bind to the ATP binding site and C-terminal myristoyl group binding site, respectively [35, 36]. ATP-competitive kinase inhibitors such as imatinib/STI571, inhibit several tyrosine kinases in addition to Abl and Arg, and were recently shown to induce formation of B-RAF/C-RAF dimers leading to ERK activation in cancer cells expressing oncogenic RAS [37]. In contrast, allosteric inhibitors such as GNF2, which target the unique Abl/Arg myristate-binding site and function as non-ATP-site and mono-selective inhibitors of the Abl kinases [38], do not target B-RAF/C-RAF and do not promote paradoxical ERK activation. Moreover, STI571 and GNF2 do not inhibit the Met receptor kinase [36, 39]. Transient treatment of MDCK cell clusters with either STI571 or GNF2 inhibited HGF-induced MDCK cell scattering, which is detected by disruption of E-cadherin-positive cell-cell junctions (Fig 1B and 1C). To ascertain whether the effects of STI571 and GNF2 were specifically mediated by the Abl kinases, Abl and/or Arg were depleted by miRNA-mediated knockdown. Loss of both Abl and Arg proteins, and to a lesser extent, Arg depletion alone, resulted in disruption of adherens junctions in MDCK cell colonies, which is consistent with our previous finding for a requirement of Abl family kinases in epithelial cell-cell junction formation and maintenance (S1A Fig) [30]. In contrast, single knockdown of Abl alone did not disrupt cell-cell contacts in the absence of HGF (Fig 1D and 1E). However, loss of Abl expression alone markedly decreased HGF-induced scattering of MDCK cell islands (Fig 1D–1F). These data support a role for Abl activity in the regulation of HGF/Met-induced cell scattering.

### Activated Abl Kinases Interact with the Met Receptor and Promote Its Phosphorylation

**Fig 2:**
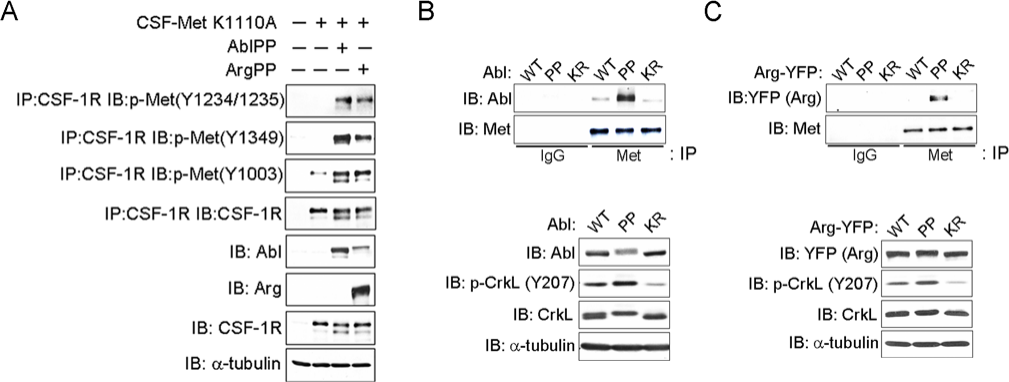
Activated Abl kinases promote Met phosphorylation and interact with Met. (A) 293T cells were transfected with vector control or a chimeric receptor (CSF-Met K1110A) comprised of the extracellular domain of CSF1R fused to the intracellular domain of kinase-inactive Met (K1110A) receptor, in the absence or presence of constitutive active AblPP or ArgPP. Cells were lysed, the lysates were incubated with anti-CSF1R antibody, and the immunoprecipitates were analyzed by western blotting with the indicated antibodies. (B-C) 293T cells were transfected with the indicated forms of Abl (B) or Arg (C). Cells were lysed after 48 hours and the endogenous Met receptor was immunoprecipitated. Met receptor-bound Abl (B) and Arg (C) proteins were analyzed by western blotting (upper panels). Expression and activity (p-CrKL) of Abl and Arg kinases in total cell lysates were analyzed by western blotting with the indicated antibodies (lower panels). Tubulin was used as loading control in all panels.

HGF induces Met dimerization and activation leading to auto-phosphorylation of tyrosine residues in the Met kinase domain (Y1234, 1235), followed by phosphorylation of the carboxy-terminal substrate-binding sites (Y1349 and Y1356), and Y1003, the binding site for the CBL E3 ubiquitin ligase, which promotes ubiquitination and degradation of the Met receptor [2, 6]. Thus, we evaluated the time course of HGF-induced Met tyrosine phosphorylation in control and Abl/Arg-depleted MDCK cells. In control MDCK cells maximal Y1232/Y1235 phosphorylation was detected at 10 min and maximal phosphorylation on Y1349 and Y1003 was at 40 min of HGF treatment (Fig 1A). Knockdown of both Abl and Arg or treatment with STI571 decreased HGF-induced Met tyrosine phosphorylation (Fig 1A and S2B Fig). However, knockdown of Abl alone did not affect Met tyrosine phosphorylation in response to HGF (S2A Fig). To assess whether Abl kinases could promote tyrosine phosphorylation of a kinase-inactive Met receptor, we employed a chimeric receptor (CSF-Met K1110A) comprised of the extracellular domain of the colony stimulating factor 1 receptor (*CSF1R*) fused to the intracellular domain of the Met receptor with a K1110A mutation that abrogates Met kinase activity and auto-phosphorylation [40]. Constitutive active Abl kinases, Abl-PP and Arg-PP, promoted tyrosine phosphorylation of the kinase-inactive Met receptor at multiple sites involved in activation and signaling (Y1234/Y1235, Y1349), as well as downregulation (Y1003) (Fig 2A). We previously showed that activated Abl kinases phosphorylate and form complexes with the platelet-derived growth factor (PDGF) receptor [41, 42]. Thus, similar to the PDGF receptor, active Abl kinases might not only promote Met tyrosine phosphorylation, but may also interact with the Met receptor. To examine this possibility we expressed either wild-type (WT), constitutively-active (PP) or kinase-defective (KR) mutant forms of Abl or Arg in 293T cells and evaluated binding to the Met receptor (Fig 2). We found that activated Abl-PP and Arg-PP preferentially bound to the Met receptor compared to WT and kinase-defective forms of Abl and Arg (Fig 2B and 2C). These results suggest that the activation status of the Abl kinases may regulate their association with the Met receptor. Consistent with this possibility, we found that while wild-type Arg fused to YFP (Arg-YFP) exhibited a uniform cytoplasmic distribution in un-stimulated MDCK cells, HGF treatment promoted co-localization of Arg-YFP with endogenous Met at membrane protrusions and some cell-cell junctions (S1B Fig). These data suggest that activation of the Met receptor may promote its association with activated Arg. Taken together, these data support the induction of bidirectional signaling between Met and Abl kinases.

### Abl Kinases Regulate Actomyosin Contractility Downstream of the Met Receptor

**Fig 3:**
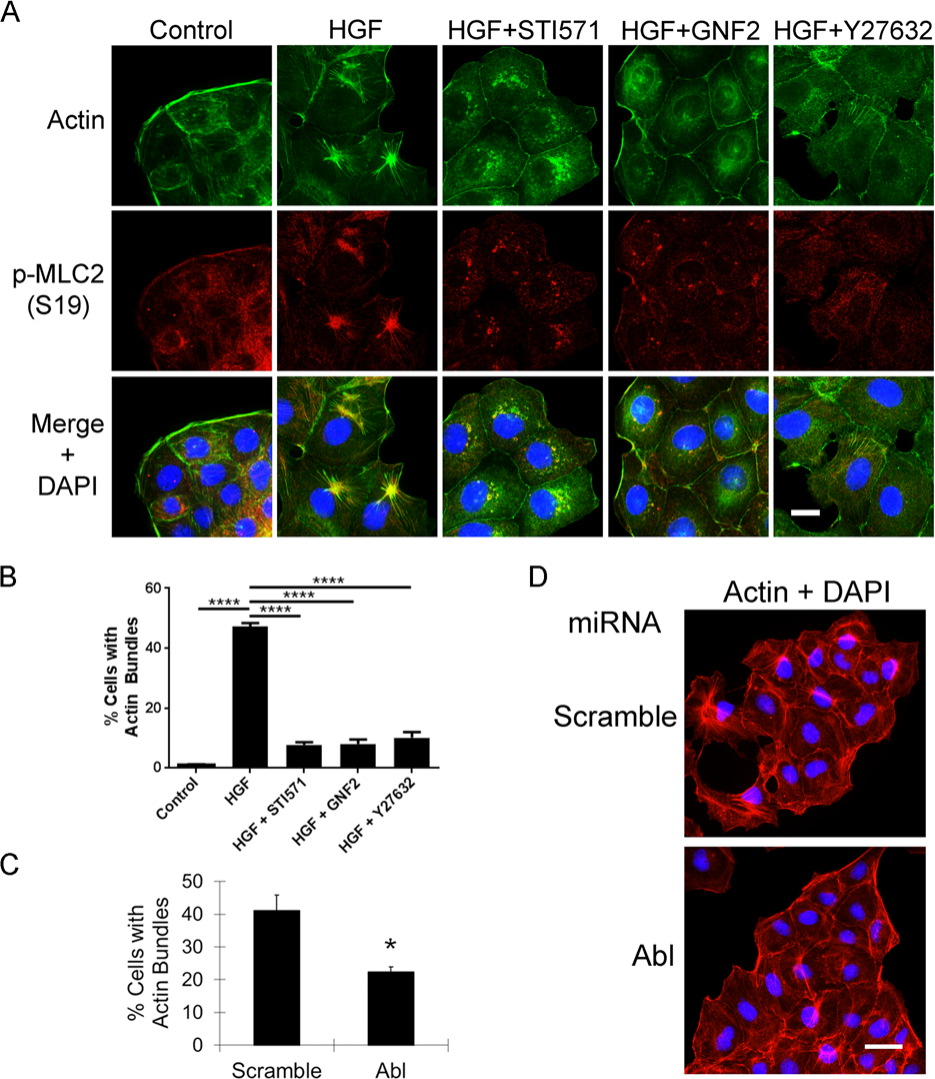
Inhibition of Abl kinases function suppresses HGF-induced actomyosin contractility. (A-B) Small molecule inhibitors of the Abl kinases suppress HGF-induced actomyosin contractility. Serum-starved MDCK cells were left untreated or treated with 20ng/ml HGF for 3 hours with the presence or absence of Abl kinase inhibitors STI571 (15μm) and GNF2 (20μm), or the ROCK inhibitor Y27632 (15μm). Cells were stained for actin and phospho-myosin light chain (p-MLC2 on S19), followed by fluorescence microscopy analysis (A). Scale bar, 10μm. The percentages of actin bundle-containing cells were quantified and analyzed by one-way ANOVA followed by Bonferroni post-test. ****P<0.0001. Error bars represent mean (n = 3) ± S.E.M. (B). (C-D) Knockdown of Abl kinase impairs HGF-induced actin bundle formation. Serum-starved MDCK cells expressing either scramble control or Abl-specific miRNA were treated with HGF for one hour, followed by staining for actin (red) and visualization by fluorescence microscopy (D). Scale bar, 20μm. The percentages of actin bundle-containing cells from each experimental group were quantified and analyzed by two-tailed unpaired Student’s t-test (C). *P<0.05. Error bars represent mean (n = 3) ± S.E.M.

HGF treatment has been shown to induce thick actin bundles which are distinct from actin stress fibers in quiescent MDCK cells [43]. Thick actin bundles together with myosin light chain generate contractile forces along actomyosin fibers and promote disruption of cell-cell junctions [44]. Because we found that inhibition of Abl kinases suppressed HGF-induced MDCK cells scattering, we evaluated whether Abl kinases might function to link Met activation to actomyosin contractility. In the absence of HGF stimulation, MDCK cells predominantly exhibited thin intracellular stress fibers and strong cortical fibers, and HGF treatment induced the formation of thick actin bundles radiating to the cell periphery (Fig 3A). The actin bundles co-localized with phospho-myosin light chain in the presence of HGF generating distinctive star-shaped structures (Fig 3A, merged bottom panel). Notably, these HGF-induced actomyosin bundles were markedly inhibited by the Abl kinase inhibitors STI571 and GNF2 (Fig 3A and 3B). Further, pharmacological inhibition of the Abl kinases decreased HGF-mediated phosphorylation of myosin light chain (p-MLC2) in MDCK cells (S3A Fig). Thus, Abl kinases modulate both MLC phosphorylation and the co-localization of myosin with actin in MDCK cells. The Rho GTPase can be activated downstream of the Met receptor, and inhibition of Rho or its effector Rho kinase (ROCK) decreases actin bundle formation and suppresses HGF-induced cancer cell spreading [44, 45]. Conversely, Rho activation by overexpression of the myosin-interacting guanine nucleotide exchange factor (MyoGEF) elicits formation of actin-myosin bundles [46]. We found that the formation of thick actin bundles after HGF treatment was significantly suppressed by the ROCK inhibitor Y27632 to a similar extent as that suppressed by the Abl kinase inhibitors (Fig 3A and 3B). Further, depletion of the Abl kinase alone resulted in 50% reduction of thick actin bundles in MDCK cells after HGF treatment (Fig 3C and 3D). These data identify new roles for Abl kinases in the formation of HGF-induced actomyosin bundles, as well as regulation of MLC2 phosphorylation in epithelial cells.

### Active Abl Kinases Promote Cell Scattering and Actomyosin Contractility in the Absence of HGF Treatment

**Fig 4:**
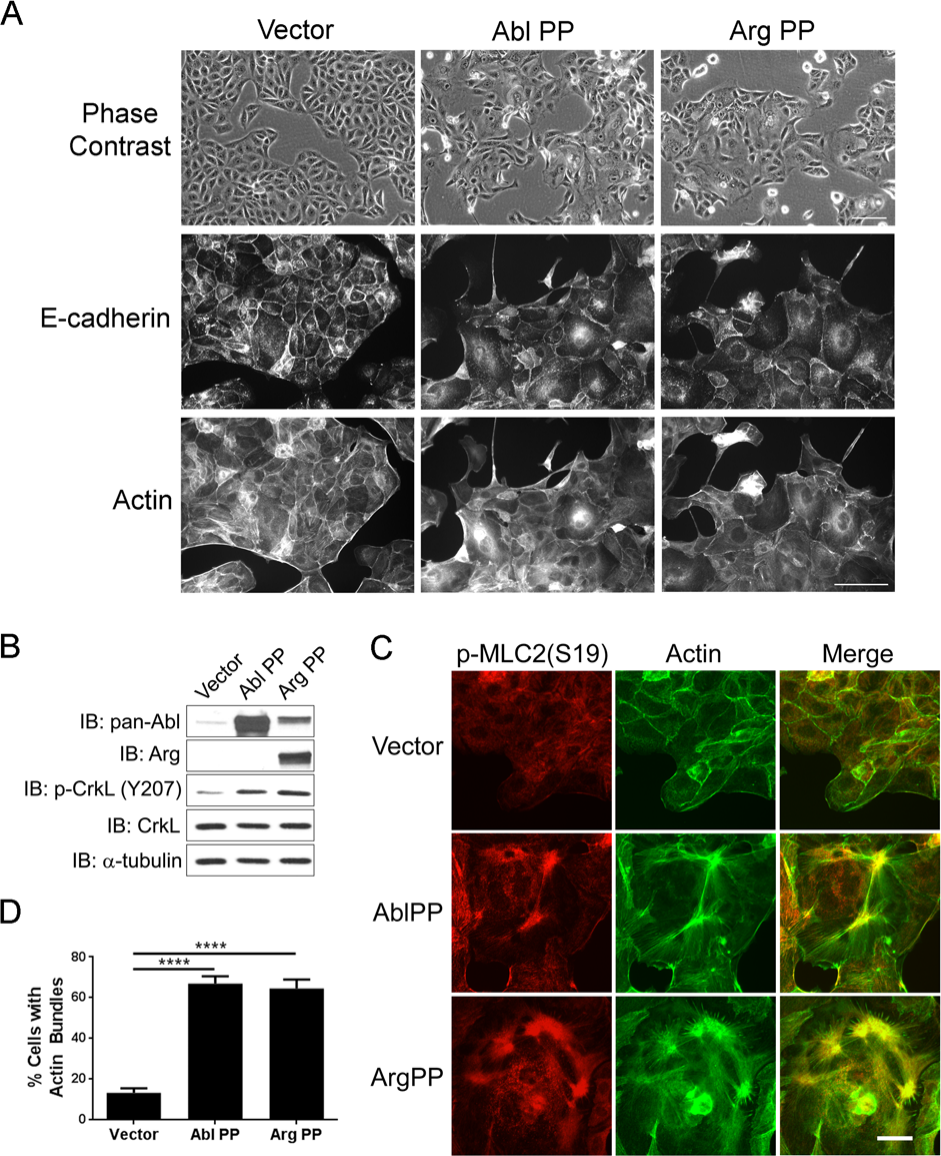
Constitutive active Abl and Arg kinases promote cell scattering and actomyosin contractility in the absence of Met activation. (A-B) MDCK cells expressing the indicated cDNAs were fixed and phase contrast pictures were acquired, followed by staining for E-cadherin and actin (A). Protein expression and activity (p-CrKL Y207) of AblPP and ArgPP kinases were analyzed by western blotting (B). (C-D) Constitutive active Abl and Arg kinases promote actomyosin contractility. MDCK cells expressing empty vector, AblPP, or ArgPP, were fixed and stained for p-MLC2 and actin, followed by fluorescence microscopy analysis (C). Scale bar 20μm. The percentages of actin bundle-containing cells from each experimental group were quantified and analyzed by one-way ANOVA followed by Bonferroni post-test. ****P<0.0001. Error bars represent mean (n = 3) ± S.E.M. (D).

To evaluate whether constitutively active Abl and Arg kinases could mimic HGF-induced phenotypes, we expressed active mutant forms of either Abl (Abl-PP) or Arg (Arg-PP) in MDCK cells. Expression of these proteins was confirmed by western blotting and resulted in enhanced phosphorylation of CrkL Y207, indicative of Abl/Arg activation (Fig 4B). Expression of either Abl-PP or Arg-PP was sufficient to promote weakening of adherens junctions and disruption of E-cadherin-positive cell-cell contacts similar to the phenotypes induced by HGF treatment in MDCK cells (Fig 4A). Some epithelial cells expressing the activated Abl kinases cells exhibited migratory, mesenchymal morphology, suggesting a partial EMT (Fig 4A). Moreover, expression of constitutively active Abl kinases induced formation of thick actin bundles that co-localized with phosphorylated myosin light chain (p-MLC), phenocopying the effects induced by HGF treatment (Fig 4C and 4D). Similarly, expression of constitutively active forms of the Abl kinases, Abl-PP and Arg-PP, markedly enhanced p-MLC2 levels in MDCK cells (S3B Fig). Taken together, these data support a role for Abl kinases in promoting Met receptor-mediated cell scattering through the regulation of actomyosin contractility.

### Inhibition of Abl Kinases Impairs HGF-induced Rho Activation

**Fig 5:**
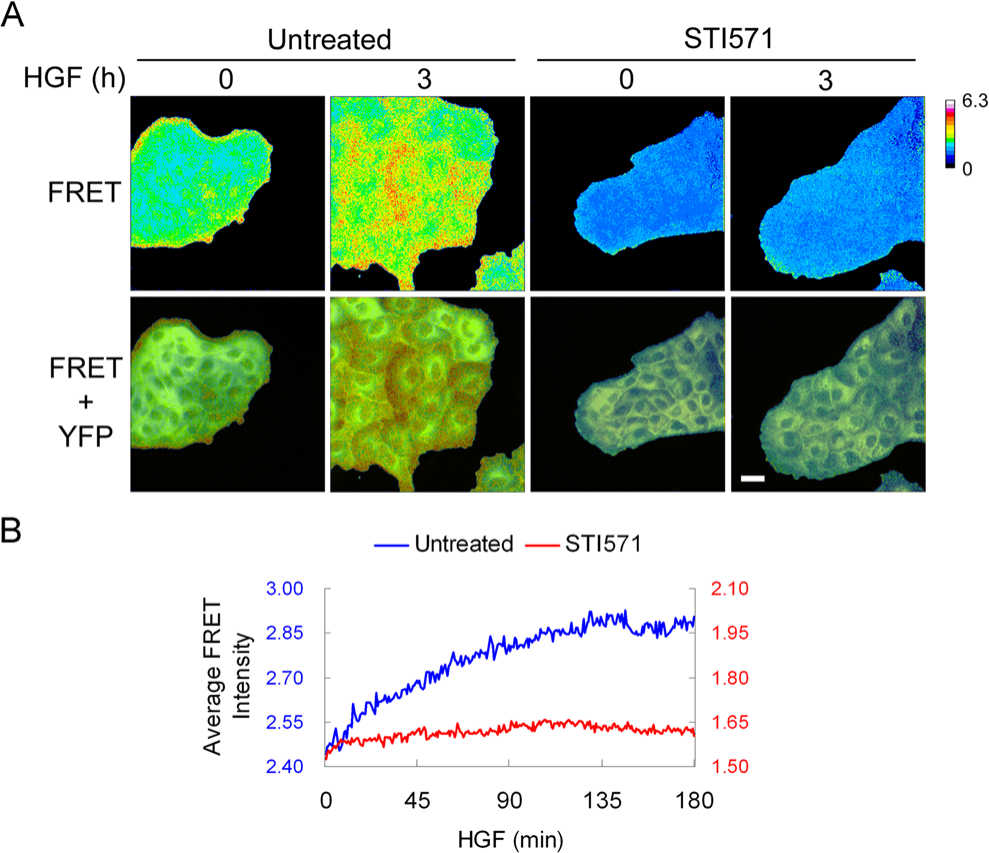
Inhibition of Abl kinases suppresses HGF-induced RhoA activation. (A) MDCK-FRET cells were grown in medium without doxycycline to induce the expression of the RhoA FRET reporter. Cells were serum-starved overnight and treated with HGF (50 ng/ml) for 3 hours in the presence or absence of 10μm STI571. Images of different channels were acquired and data were analyzed using MetaMorph software. The FRET signal reflecting RhoA activity is shown. The YFP signal is used to define cell bodies. (B) Quantification of the FRET signal over time from each experimental group in (A) is shown.

Similar to the inhibitory effect of inactivation of Abl kinases, inhibition of ROCK activity suppressed HGF-induced formation of thick actin bundles (Fig 3A and 3B). Because ROCK can be activated downstream of the active RhoA GTPase, we evaluated whether Abl kinases could directly regulate RhoA activity downstream of the Met receptor. To evaluate whether Abl kinases regulate the spatio-temporal activation of Rho in epithelial in response to HGF, we used fluorescence resonance energy transfer (FRET) of a reporter for the RhoA GTPase in MDCK cells (MDCK-FRET) [47]. Prior to HGF treatment, RhoA activity was detected in the peripheral region of epithelial cell clusters, with low intracellular, cytosolic RhoA activity (Fig 5A). This finding is in agreement with the reported presence of actin stress fibers in the periphery of cell clusters and requirement for RhoA activity for the maintenance of these cortical fibers [43, 45]. HGF treatment induced cell motility and enhanced RhoA activity in the lamellipodia of moving cells (S1 Movie). A gradual increase in RhoA activity was also observed intracellularly within the cell clusters, and co-localized with thick actomyosin bundles (Fig 5A). Importantly, HGF-induced increase in RhoA activity was markedly inhibited by STI571 (Fig 5A, 5B and S2 Movie). Treatment with the Abl allosteric inhibitor GNF2 similarly inhibited localized RhoA activation (S4 Fig, S3 and S4 Movies). Taken together, these data demonstrate that the activity of the Abl kinases is required for RhoA activation and regulation of RhoA-mediated actomyosin contractility downstream of the ligand-activated Met receptor in MDCK epithelial cells.

### Inactivation of Abl and Arg Inhibits HGF-induced Tubulogenesis

**Fig 6:**
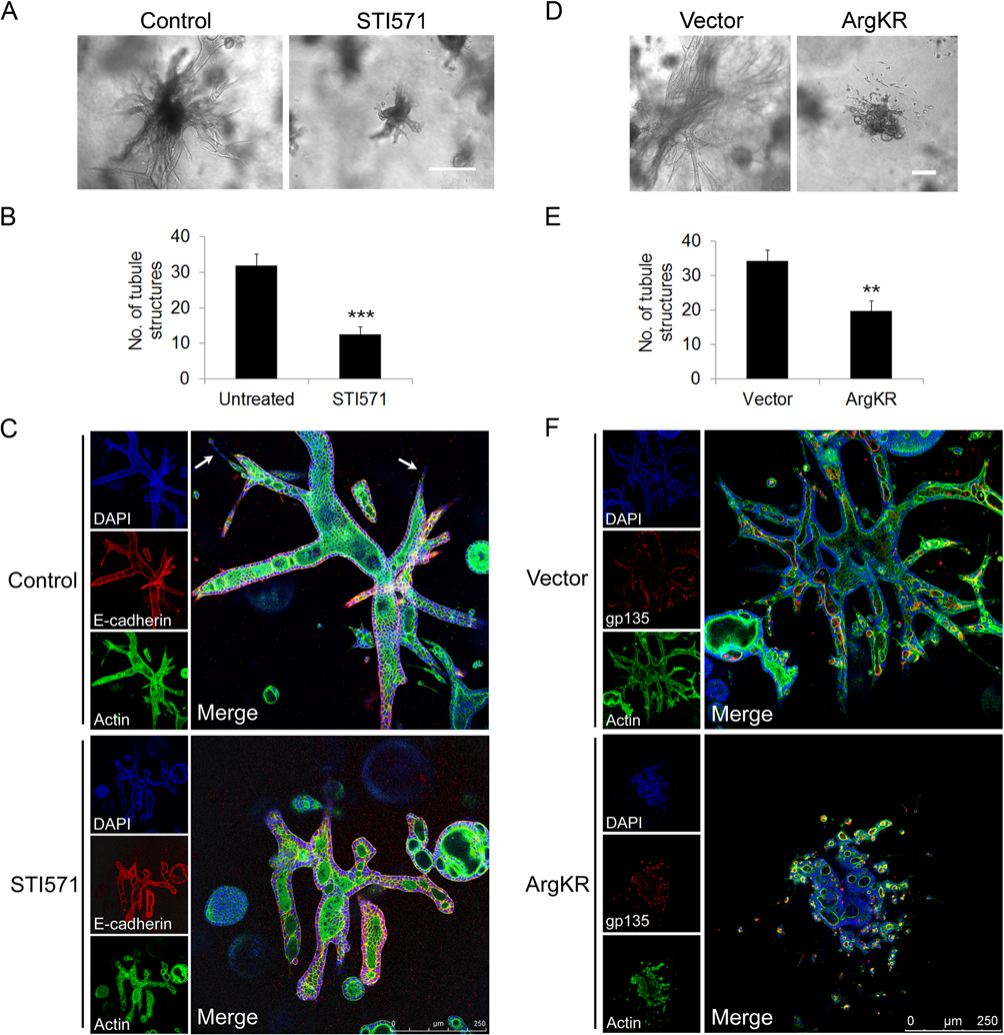
Inactivation of Abl kinases impairs HGF-induced tubulogenesis. (A) MDCK type II cells were grown in collagen gels for 10 days and then treated with HGF (20 ng/ml) for another 7 days with or without 10μM STI571. The gels were then fixed and visualized by microscopy; bright field pictures of tubule structures are shown. (B) The number of tubule structures per sample from each experimental group was quantified and analyzed by two-tailed unpaired Student’s t-test. ***P<0.001. Error bars represent mean ± S.D. (C) 6-day MDCKII cysts were treated with HGF for 10 days with or without STI571. Gels were fixed and analyzed for the indicated markers by confocal microscopy. Scale bar, 250μm. (D) MDCK type II cells expressing either vector or dominant-negative ArgKR mutant were grown in collagen gels for 10 days. Cysts were treated with HGF for another 5 days. After treatment, gels were fixed and visualized by microscopy; bright field pictures of tubule structures are shown. (E) The number of tubule structures per sample was quantified and analyzed by two-tailed unpaired Student’s t-test. **P<0.004. Error bars represent mean ± S.D. (F) Gels were fixed and analyzed for the indicated markers by confocal microscopy. Scale bar, 250μm.

Signaling from the Met receptor is known to induce EMT and promote formation of epithelial tubules and invasive growth. To evaluate whether Abl kinases play a role in Met-regulated EMT and invasive growth, we used a three-dimensional (3D) epithelial culture system. MDCK type II (MDCKII) cells were grown in a 3D collagen matrix to form cyst structures with a hollow lumen surrounded by a single layer of polarized cells [48]. Treatment of cysts with HGF promoted development of epithelial tubule structures, in a process known as tubulogenesis, which requires coordinated regulation of EMT, cell migration and invasion [49]. Treatment with STI571 decreased HGF-induced formation of 3D epithelial tubules, with greater than a two-fold reduction in the number of tubule structures in the STI571-treated cultures (Fig 6A and 6B). Tubules in the control group exhibited sharp tips, whereas STI571-treated tubules presented bulky and rounded tips. Sharp tubule tips result from a single chain of migratory cells undergoing partial EMT induced by HGF (arrows in Fig 6C). The rounded tubule tip structures in the STI571-treated cells suggest inhibition of HGF-induced partial EMT and cell invasion. Further, expression of a kinase-defective mutant of Arg (Arg-KR) impaired tubule formation (Fig 6D and 6E). Constitutive expression of kinase-defective Arg inhibits branching tubules to a greater extent than transient STI571 treatment (Fig 6F). This effect may be due to disruption of cyst polarity by kinase-defective ArgKR at early stages of cyst development and/or dissolution of cell-cell contacts in the branching tubules leading to the appearance of single cells or cell clusters [21].

### Abl Kinases Are Activated in Met-driven Mouse Mammary Tumors and Abl Inhibitors Suppress HGF-induced Migration and Invasion of Breast Cancer Cells

**Fig 7:**
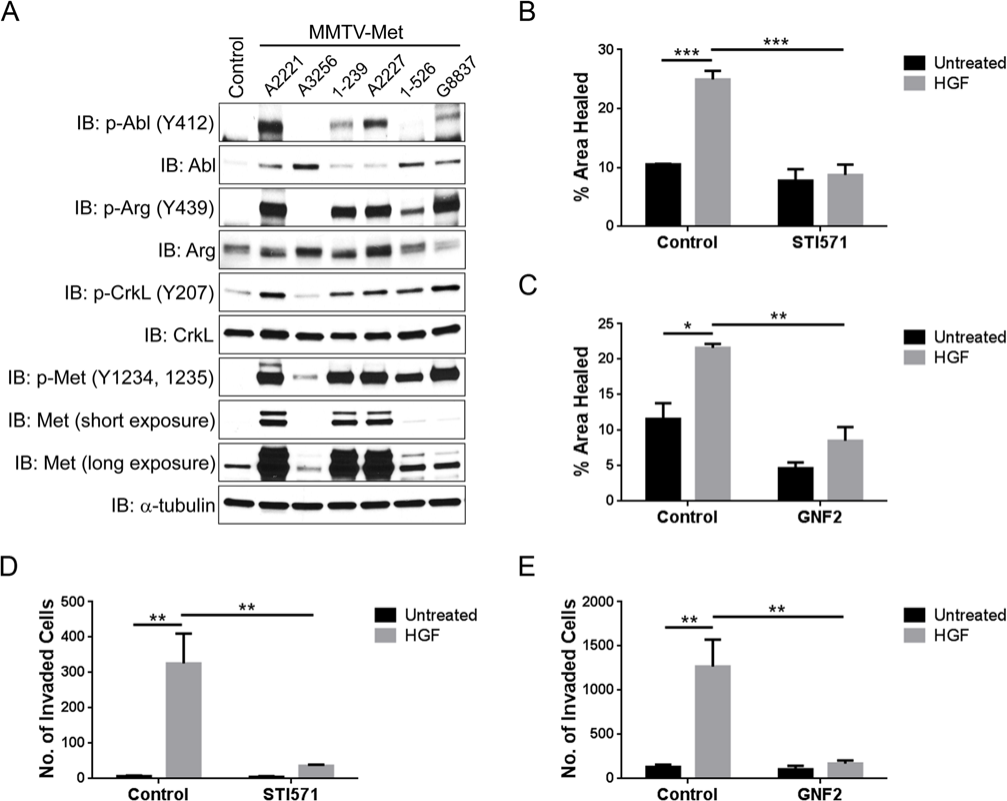
Abl kinases are activated in Met-driven mouse mammary tumors and inhibition of Abl kinases suppresses HGF-induced migration and invasion of breast cancer cells. (A) Cell lines derived from mammary tumors isolated from mice expressing oncogenic Met receptor driven by the MMTV promoter (MMTV-Met) (lanes 3, 6, and 7) or MMTV-Met; Trp53fl/+; Cre+ mice (lanes 2, 4, and 5), were lysed and subjected to western blotting with the indicated antibodies. Mammary tissue from a wild-type female mouse was used as a control (lane 1). (B-C) Inhibition of Abl kinases suppresses HGF-induced breast cancer cell migration. A wound was generated within a confluent layer of serum-starved MDA-MB-231 cells. Cells were pre-treated with either STI571 (B) or GNF2 (C) for several hours and then allowed to migrate in media as indicated for 16h. Bright field pictures were acquired and the images were analyzed with ImageJ. The percentages of wound area healed by cell migration were quantified and analyzed by two-way ANOVA followed by Bonferroni post-test. ***P<0.001; **P<0.01; *P<0.05. Error bars represent mean (n = 3) ± S.E.M. (D-E) HGF-induced breast cancer cell invasion was decreased by Abl kinase inhibitors. Serum-starved MDA-MB-231 cells were plated in the upper well of the matrigel invasion chambers in the presence or absence of either STI571 (D) or GNF2 (E). HGF was added in the lower chambers with or without the Abl kinase inhibitors. 48 hours later, invaded cells were quantified and analyzed by two-way ANOVA followed by Bonferroni post-test. **P<0.01. Error bars represent mean (n = 3) ± S.E.M.

Hyper-activation of the HGF-Met pathway correlates with aggressiveness and poor prognosis of diverse carcinomas including breast cancers [50]. We first examined the activity of Abl kinases in mouse mammary tumors derived from mice harboring a weakly oncogenic variant of *Met* under the control of the murine mammary tumor virus (MMTV-Met) [51], as well as MMTV-Met mice harboring conditional deletion of *Trp53* in the mammary glands [52]. Loss of p53 in the MMTV-Met tumors is associated with genomic amplification of the endogenous Met locus and elevated Met protein expression [52]. We found that tumors with active Met signaling had increased activities of Abl and Arg kinases, as shown by enhanced tyrosine phosphorylation of the Abl kinases with Abl/Arg phospho-specific antibodies as well as hyper-phosphorylation of CrkL on the Abl/Arg-specific site (Y207) (Fig 7A). The tumors with the highest levels of active, phosphorylated Met (p-Met Y1234/1235) displayed the highest levels of active Abl and Arg regardless of p53 status. Consistent with these findings, treatment of human breast cancer MDA-MB-231 cells with HGF induced activation of Abl kinases as measured by phosphorylation of CrkL Y207, and this phosphorylation was blocked by the Abl inhibitor STI571 (S5A Fig). Moreover, inhibition of Abl kinases with either STI571 or GNF2 decreased HGF-induced MDA-MB-231 breast cancer cell migration in a wound healing assay (Fig 7B, 7C and S5C Fig). Further, HGF-induced invasion of MDA-MB-231 cells was also markedly impaired by both Abl/Arg kinase inhibitors (Fig 7D and 7E). The inhibitory effects of these compounds was not due to suppression of cell growth and viability as STI571 and GNF2 did not inhibit MDA-MB-231 cell growth and survival within 24 hours (S5B Fig). The phenotypes induced by inhibition of the Abl kinases were not restricted to breast cancer cells as treatment of the MDA-MB-435s melanoma cell line (also known as M14) with STI571 markedly inhibited HGF-induced invasion without inhibiting cell growth (S6 Fig). Taken together, these data indicate that Abl kinases are activated by an oncogenic Met receptor in mammary tumors, and Abl kinases inhibitors suppress invasion and migration of cancer cells driven by activated Met signaling.

## Discussion

Activation of HGF-Met signaling induces diverse morphogenetic responses including formation of branching tubules, cell scattering, and invasion. Here we identified a new role for the Abl family of non-receptor tyrosine kinases in the regulation of these HGF/Met morphogenetic processes. We found that knockdown or pharmacological inhibition of the Abl kinases markedly decreased HGF-induced cell scattering, actomyosin bundles and branching tubule formation in MDCK epithelial cells. Conversely, expression of constitutive active forms of the Abl kinases mimicked HGF-induced formation of thick actin bundles and actomyosin contractility.

A number of proteins targeted by the Abl kinases have been functionally linked to the activated Met receptor. Among these are the Crk/CrkL adaptors, the matrix metalloproteinase MT1-MMP, and β1-integrin [21–23, 53–55]. These molecules have been implicated in the regulation of HGF/Met morphogenetic processes such as cell polarization, scattering, and tubulogenesis [56, 57]. Unexpectedly, we found that Abl kinases link HGF-induced Met activation to the activation of the RhoA GTPase in MDCK epithelial cells. The Cdc42 and Rac GTPases were previously shown to be activated in response to HGF in MCDK cells, and the activity of the Cdc42/Rac-regulated p21-activated kinase (PAK) was required for epithelial cell spreading [34]. Here we demonstrate that Abl kinases are required for HGF/Met-induced RhoA activation and regulation of actomyosin contractility in a spatio-temporal manner. The activity of the Abl kinases is required for HGF-induced RhoA activation at dynamic lamellipodia structures, and also for the gradual RhoA activation in the cytosol leading to induction of actomyosin-rich bundles. In contrast to the findings in MDCK cells, we and others previously reported that Abl kinases negatively regulate RhoA activity in other cell types such as fibroblasts, neurons and rat epithelial cells [30–32, 58–60]. Inhibition of Abl kinases in confluent NBTII rat epithelial cells induced RhoA activity and disrupted adherens junctions [30]. Thus, modulation of RhoA activity by Abl kinases is cell context dependent and may depend on the presence of distinct signaling networks and the balance of RhoA activators and inhibitors in various cell types and tissues. Consistent with this possibility, STI571 treatment of thyroid cancer cells and HeLa cells was reported to increase cell migration in response to HGF [61, 62]. However, it is unclear whether the effects of STI571 on cell migration of these cells were mediated by Abl kinases or by other STI571-targeted kinases due to the lack of knockdown and gain-of—function approaches to specifically target Abl and Arg kinases in these studies.

In addition to regulating morphogenetic processes and other pathways dependent on dynamic remodeling of the cytoskeleton, the Abl kinases have been reported to regulate cell proliferation and survival depending on the cellular context [24]. Abl kinases were shown to regulate cell proliferation, anchorage-independent growth and survival of breast cancer and melanoma cells [28, 29]. Further, it was reported that c-Abl (*ABL1*) can function downstream of the Met receptor to regulate survival and growth of gastric GTL16 and liver HepG2 cells, and these effects were dependent on the presence of wild type p53 [63]. The c-Abl kinase was also reported to be activated downstream of Ron, a receptor tyrosine kinase related to the Met receptor, and Ron-induced c-Abl activation promoted breast cancer cell proliferation [64]. In contrast, we found that inhibition of Abl kinases did not affect the growth and viability of the cancer cells analyzed. It is unclear whether the differential requirement for Abl kinases for cell growth and viability is due to the distinct stimuli or assay conditions employed, or to the existence of differential signaling networks driving cell growth and survival in cell context dependent manner.

Abl kinases are activated downstream of multiple RTKs including the EGFR, PDGFR, and Tie2 [41, 65, 66]. Once activated, the Abl kinases promote downstream signaling by targeting multiple pathways required for morphogenesis, growth and/or survival. Notably, we have previously reported that upon activation, the Abl kinases can target the activating RTKs through transcriptional and post-transcriptional mechanisms [41, 65, 66]. In this regard, we found that upon activation by ligand-activated EGFR and PDGFR, Abl kinases can induce phosphorylation of specific tyrosine residues on both RTKs. For example, Abl-mediated phosphorylation of EGFR Y1173, inhibits CBL-dependent EGFR degradation [65]. Here we found that upon activation downstream of HGF/Met, Abl kinases can promote the phosphorylation of Met at multiple tyrosine residues (Y1349) required for engaging downstream signaling pathways, as well as phosphorylation of sites (Y1003) implicated in the termination of Met signaling. Thus, Abl kinases may function to transiently activate Met in a spatio-temporal restricted manner. Previous studies have suggested a positive role for the Src kinase in regulating Met phosphorylation in multiple cancers such as lung, breast and bladder carcinomas [67–69]. Growth factor treatment activates both Src and Abl kinases and these kinases can engage in bi-directional, activating interactions, and may function together to potentiate Met phosphorylation and signaling [70, 71]. Our finding that active (tyrosine phosphorylated) Abl and Arg kinases preferentially form complexes with the Met receptor, suggest that upon activation, the Abl kinases interact directly or indirectly with the receptor to enhance and subsequently terminate Met signaling in MDCK cells. In this regard, an earlier report identified c-Abl (*ABL1*) as a direct binding partner of the Met receptor using a yeast two-hybrid screen, and showed that this interaction was independent of the Gab1 scaffold protein [72].

Deregulation of RTK signaling is associated with the progression of many carcinomas, and plays a role in the emergence of therapy-resistance [73, 74]. Among the mechanisms that confer resistance is the upregulation of alternative RTKs in various tumors [74–76]. HGF promotes resistance to drugs targeting HER2 in breast cancer and BRAF in melanoma cell lines [75]. In Her2-overexpressing breast cancer cells, treatment with trastuzumab leads to upregulation of Met expression, which contributes to trastuzumab resistance [77]. Met activation also confers resistance to the EGFR inhibitor gefitinib through Src activation [78]. In Met-amplified lung cancer cells, activation of EGFR signaling contributes to the development of drug resistance after prolonged inhibition of the Met receptor [79]. These and other studies have suggested that combination therapy simultaneously targeting multiple RTKs is necessary to effectively inhibit tumor progression and prevent development of drug resistance [75, 76]. Because the Abl family kinases are activated downstream of multiple RTKs, targeting Abl kinases with specific inhibitors could disrupt signaling networks persistently activated in some solid tumors leading to invasion, dissemination, and in some instances even growth and survival. Future studies are needed to identify cell types that rely on hyperactive Abl kinases to promote invasion and other morphogenetic processes in response to HGF/Met-dependent and—independent signaling during normal development and cancer.

## Materials and Methods

### Antibodies and Reagents

Mouse anti-Abl antibody was from BD Bioscience. Mouse anti-Arg antibody, rabbit anti-CrkL antibody, rabbit anti-Met antibody, and rat anti-CSF-1R were from Santa Cruz Biotechnology. Phospho-specific antibodies for Abl (pY412) (BD) and Arg (pY439) (ABGENT) were used for western blotting. Mouse anti-tubulin antibody was from Sigma. Rabbit anti-phospho-CrkL (Y207) antibody, rabbit anti-Met (Y1234/1235) antibody, rabbit anti-Met (Y1349) antibody, rabbit anti-Met (Y1003) antibody, mouse anti-phospho-MLC2 (S19) antibody were from Cell Signaling Technology. Rat anti-E-cadherin was from Zymed (Invitrogen). Mouse anti-gp135 antibody was a gift from Dr. George Ojakian (SUNY Downstate Medical Center). Mouse anti-GFP antibody and Fugene 6 were from Roche. Hepatocyte growth factor (HGF) and GNF2 were from Sigma. pBabe-Sin-Puro-Tet-RhoA Biosensor (RhoA FRET reporter) was from Addgene. HGF used for 3D tubulogenesis was from ProSpec. OxyFluor used in live cell imaging was from Oxyrase Inc. Portein G sepharose was from GE healthcare. ProLong antifade mounting media was from Invitrogen.

### Cell Lines and Cell Culture Conditions

MDCK epithelial cells, HEK-293T cells, MDA-MB-231 breast cancer cells, and MDA-MB-435s melanoma cell line [80] were cultured in Dulbecco's modified Eagle's medium (DMEM) supplemented with 10% fetal bovine serum (FBS) (Invitrogen). MDCK type II (MDCKII) cells used in 3D tubulogenesis assays were cultured in MEM supplemented with 5% heat-inactivated FBS (Invitrogen). MDCK Tet-Off cells were from Clontech and were cultured in DMEM (low glucose) supplemented with 10% FBS (Clontech) and 1μg/ml doxycycline. Cells were maintained at 37°C and 5% CO2.

Cell lines were derived from mouse mammary tumors harboring MMTV-Met [51] or MMTV-Met; Trp53 fl/+; Cre [52]. The identity of the cell lines used is as follows: A2221 was derived from tumor A1005 (MMTV-Met;Trp53fl/+); A3256 was derived from tumor 7325 (MMTV-Met); 1–239 was derived tumor A1129 (MMTV-Met;Trp53fl/+); A2227 was from tumor A1471 (MMTV-Met;Trp53fl/+); 1–526 was from tumor 4691 (MMTV-Met); and G8837 was from tumor 5156 (MMTV-Met).

### Transfection and Retroviral Transduction

293T cells were transfected with either PK1 vector, PK1-AblPP, PK1-ArgPP and/or RhoA FRET reporter together with gag/pol and VSVG packaging plasmids (Fugene 6). After 24 hours, the medium was removed and cells were supplemented with fresh medium and incubated for another 24 hours. The virus-containing medium was incubated with target cells in the presence of polybrene (4μg/ml) for 48 hours. Cells were selected with either 1μg/ml puromycin or by sorting for the YFP positive population with fluorescence-activated cell sorting (FACS).

### Gene Silencing of Abl, Arg Kinases with Lentiviral-derived miRNA Mimics

Lentiviral transduction of Abl/Arg micro-RNAs (miRNAs) has been described elsewhere [81–83]. Cells expressing miRNAs were selected by FACS for the GFP-positive population. Abl miRNA sequence: GGTGTATGAGCTGCTAGAGAA. Arg miRNA sequence: AGGTACTAAAGTGGCTCTGAG.

### Immunoprecipitation

Cells were washed with PBS and incubated in lysis buffer (50mM Tris pH 7.5, 150mM NaCl, 1% NP40) with protease inhibitors on ice for 30 minutes. Cell lysates were centrifuged at 20,000g at 4°C for 15 minutes, and protein lysates (1 mg) were incubated with antibody (2μg) at 4°C for 4 hours, followed by incubation with protein G sepharose at 4°C for another 4 hours. Beads were washed with lysis buffer three times and suspended in SDS sample buffer, followed by western blotting.

### 3D Tubule Formation and Quantification

MDCKII cells were cultured in 3D collagen as previously described [84]. In brief, liquid collagen media was prepared by mixing media components (750μl 200mM L-Glutamine, 196μl 7.5% NaHCO3, 625μl 10x MEM and 125μl 1M HEPES) with 4.13ml purified bovine collagen solution (Advanced Biomatrix 5005-B) and adjusted to pH 7.5 with 1M NaOH. MDCKII cells were trypsinized into single cell suspension and mixed with collagen (20,000 cells/ml); 200μl of cell-collagen mixture was added into each membrane insert (NUNC 136935) placed in a 24-well plate. The cell-collagen mixture was incubated at 37°C for an hour to allow the gel to solidify, followed by addition of 0.5ml culture media to the inner well of the chamber and 1ml to the outer well. Growth medium was replenished every 3 days. After 6–10 days, cysts were treated with HGF (20ng/ml) for another 5–10 days to induce tubule formation, and quantified under a bright field microscope (2.5x objective). The total number of tubule structures in each collagen gel was counted and results from multiple independent experiments were subjected to statistical analysis.

### Immunofluorescence of 3D Epithelial Tubules

A modified version of a previously described protocol was used for immunofluorescence staining of 3D epithelial tubules [84]. In brief, the gel in the membrane insert was washed once with PBS and incubated with collagenase solution (0.1kU/ml in PBS) at 37°C for 10 minutes. The gel was then washed once with PBS and fixed in 4% PFA for 30 minutes at room temperature. After washing the gel with PBS, residual PFA in the gel was quenched with 75mM NH4Cl and 20mM glycine (pH 8) in PBS at room temperature for 30 minutes. The gel was then incubated with permeabilization solution (0.5–1% Triton X-100 and 0.7% fish skin gelatin in PBS) at room temperature for 30 minutes. Primary antibodies were diluted in permeabilization solution and incubated with the gel overnight at 4°C with gentle shaking, followed by washing 5 times with permeabilization solution (30 minutes each), and then incubated with fluorophore-conjugated secondary antibodies diluted in permeabilization solution for 3 hours at room temperature. After incubation, the gel was washed 5 times with permeabilization solution (30 minutes each) and then two times with PBS (5 minutes each). The gel was then taken out of the insert and mounted on the coverslide with ProLong antifade mounting media. After drying overnight, the slide was kept in the dark followed by confocal imaging.

### Induction of RhoA FRET Reporter in MDCK Tet-Off Cells

MDCK Tet-Off cells retrovirally expressing RhoA FRET reporter (MDCK-FRET cells) were maintained in growth medium containing 1μg/ml doxycycline to suppress the expression of the RhoA FRET reporter. For induction of the reporter, cells were washed once with PBS and trypsinized into a single-cell suspension. After centrifugation, the cell pellet was washed once with growth medium without doxycycline. Cells were then resuspended in the same medium and plated sparsely in 35mm culture dishes with glass bottoms (MatTek). The next day, the growth medium was removed and cells were serum-starved with low-glucose DMEM containing 0.25% Tet System Approved FBS overnight before the FRET imaging.

### Fluorescence Resonance Energy Transfer (FRET) Imaging with “Zeiss Axio Observer” Live Cell Microscope

Serum-starved MDCK-FRET cells in 35mm dishes were incubated with 2ml FRET medium (low-glucose DMEM without phenol red, 1:100 dilution of OxyFluor, 10mM lactate, 50mM Hepes and 0.25% Tet System Approved FBS) for 30 minutes on the live cell microscope chamber pre-warmed to 37°C and supplemented with 5% CO2. Images were taken in 5–10 cell-free areas in all three channels (CFP, FRET and YFP) for image analysis. Cells with medium fluorescence intensity were chosen for imaging analysis. After acquisition of three baseline images, 250ng/ml HGF diluted in 0.5ml FRET medium was added carefully to the cells, which were then imaged in all three channels every 45 seconds for 4 hours.

### FRET Image Analysis

Methods for FRET image analysis have been described elsewhere [85]. In brief, images acquired in cell-free areas at the beginning of the imaging process were averaged to create a “shading distribution” image. Images acquired in each channel were normalized against the corresponding “shading distribution” image to correct for shading (uneven illumination). After the shading correction, images were background subtracted using the plug-in module in Metamorph. Image registration was performed with the “color-align” and “subpixel shift” plug-in modules to correct pixel misalignments. Image masking was then carried out to remove the background pixels in cell free areas and the FRET signal was analyzed by dividing the FRET channel images with those of CFP channels. The resulting FRET images were presented with pseudo-colors.

### Cell Migration and Invasion Assay

For wound-healing migration assays, cells were grown in a confluent monolayer in a 6-well plate and serum-starved overnight. A wound was created with a pipette tip (P200) and cells were allowed to migrate for 16 hours. Bright field pictures of the wounds were acquired before and after the migration. Images were processed as described (http://www.le.ac.uk/biochem/microscopy/wound-healing-assay.html). For invasion assays, breast cancer cells were serum-starved overnight. Matrigel invasion chambers (BD, #354480) were hydrated in serum-free medium for 2 hours at 37°C. Cells were trypsinized into a single-cell suspension and 50,000 cells in serum-free medium with either 0.1% BSA (for experiments with STI571) or 0.1% BSA + 0.1% FBS (for experiments with GNF2) were added inside the chamber, and serum-free medium containing HGF and either 0.1% BSA (for experiment with STI571) or 0.1% BSA + 0.1% FBS (for experiments with GNF2) was added outside the chamber. Cells were allowed to invade at 37°C for 24 hours (MDA-MB-435s) or 48 hours (MDA-MB-231), followed by fixation and staining with PROTOCOL HEMA 3 STAIN SYSTEM (Fisher Scientific, 22-122-911). Bright field pictures were acquired and the number of invading cells was quantified.

### Statistical Analysis

All statistical analyses were performed using GraphPad Prism 6 software. Comparisons of two groups were performed using Student t tests (two-tailed). Comparisons involving multiple groups were evaluated using one-way or two-way ANOVA, followed by Bonferroni posttests, as indicated. For all tests, P<0.05 was considered statistically significant.

## Supporting Information

S1 Fig Arg inactivation disrupts adherens junctions and Arg is recruited to the Met receptor after HGF treatment. **(A)** MDCK cells expressing the indicated miRNAs were fixed. Bright field pictures were taken and shown in upper panels. Scale bar, 50μm. Adherens junctions were detected by staining cells with anti-E-cadherin antibody (lower panels). Scale bar, 20μm. **(B)** MDCK cells expressing low levels of wild-type Arg-YFP were serum-starved overnight and treated with HGF (20 ng/ml) for 10 minutes. Cells were fixed and stained for YFP and Met and visualized by confocal microscopy. Scale bars, 25μm.
(TIF)

S2 Fig Abl and Arg kinases modulate Met receptor tyrosine phosphorylation. **(A)** Inactivation of Abl kinase alone does not affect HGF-induced Met receptor phosphorylation. Serum-starved MDCK cells expressing either scramble control or Abl miRNA were treated with 20 ng/ml HGF for the indicated times. Cells were lysed and the lysates were subjected to western blotting with the indicated antibodies. **(B)** Pharmacological Inhibition of Abl kinases decreases Met receptor phosphorylation following HGF stimulation. MDCK cells were serum starved with 0.25% FBS for 20h prior to stimulation with 20ng/ml HGF in the presence or absence of 15uM STI571. Cell lysates were subjected to western blotting for the indicated antibodies. Treatment with 15uM STI571 decreased tyrosine phosphorylation of Y207 of CrkL as well as Y1349, 1003 and 1234/1235 residues of the c-Met receptor.
(TIF)

S3 Fig Abl kinases regulate myosin light chain phosphorylation in MDCK cells. **(A)** Inhibition of Abl kinases decreased myosin light chain (MLC) phosphorylation in MDCK cells upon HGF treatment. Serum starved MDCK cells were treated with HGF (20ng/ml) in the presence or absence of 10uM STI571. Cell lysates were subjected to western blotting for the indicated antibodies. **(B)** Active mutants of Abl/Arg kinases induced hyperphosphorylation of the myosin light chain. MDCK cells expressing either vector control, or constitutively active Abl-PP or Arg-PP were lysed and the lysates were subjected to western blotting with the indicated antibodies.
(TIF)

S4 Fig Inhibition of Abl kinases with GNF2 suppresses HGF-induced RhoA activation. **(A)** MDCK-FRET cells were grown in medium without doxycycline to induce the expression of RhoA FRET reporter. Cells were serum-starved overnight and treated with HGF (50 ng/ml) for 3 hours in presence or absence of 20μm GNF2. Images of different channels were acquired and data were analyzed using MetaMorph software. The FRET signal reflecting RhoA activity is shown. YFP signal is used to define cell bodies. Scale bar, 15μm. **(B)** quantification of the FRET signal over time from each experimental group in **(A)** is shown.
(TIF)

S5 Fig Inhibition of Abl kinases suppresses migration of MDA-MB-231 cells. **(A)** Abl kinases are activated by Met in MDA-MB-231 cells. Serum-starved MDA-MB-231 cells were treated with HGF for 30 min with or without 10μM STI571. Cell lysates were subjected to western blotting with the indicated antibodies. **(B)** MDA-MB-231 cells (5,000) were plated in each well of a 96-well plate and were left either untreated or treated with HGF, with or without Abl kinase inhibitors. After 24 hours, cells were subjected to the MTS cell viability assay, and A490 values were measured and analyzed by one-way ANOVA. Error bars represent mean ± S.D. **(C)** A wound was generated within a confluent monolayer of serum-starved MDA-MB-231 cells. Indicated cells were pre-treated with STI571 and then allowed to migrate for 16 hours as indicated. Bright field pictures were acquired and the images were analyzed with ImageJ. Scale bar, 200μm.
(TIF)

S6 Fig Inhibition of Abl kinases suppresses invasion of MDA-MB-435s cells. **(A)** Serum-starved MDA-MB-435s cells were treated with HGF for 30 min with or without 10μM STI571. Cell lysates were subjected to western blotting with the indicated antibodies. **(B)** MDA-MB-435s cells (5,000) were plated in each well of a 96-well plate and left either untreated or treated with HGF with or without STI571. After 24 hours, cells were subjected to the MTS cell viability assay and A490 values were measured and analyzed by one-way ANOVA. Error bars represent mean ± S.D. **(C)** Serum-starved MDA-MB-435s cells were plated in the upper well of the matrigel invasion chambers in the presence or absence of STI571. HGF was added in the lower chambers with or without STI571, and after 48 hours, cells invading the undersurface were quantified and analyzed by two-way ANOVA followed by Bonferroni post-test. **P<0.01. Error bars represent mean (n = 3) ± S.E.M.
(TIF)

S1 Movie HGF-induced RhoA activation (control for S2 Movie). MDCK-FRET cells were grown in medium without doxycycline to induce the expression of RhoA FRET reporter. Cells were serum-starved overnight and treated with 50 ng/ml HGF for 3 hours. Images of different channels were acquired and data were analyzed using MetaMorph and ImageJ. The FRET signal reflecting RhoA activity is shown.
(AVI)

S2 Movie Abl inhibitor STI571 suppresses HGF-induced RhoA activation. MDCK-FRET cells were grown in medium without doxycycline to induce the expression of RhoA FRET reporter. Cells were serum-starved overnight and treated with 50 ng/ml HGF for 3 hours in the presence of 10μm STI571. Images of different channels were acquired and data were analyzed using MetaMorph and ImageJ. The FRET signal reflecting RhoA activity is shown. Compare to S1 Movie.
(AVI)

S3 Movie HGF-induced RhoA activation (control for S4 Movie). MDCK-FRET cells were grown in medium without doxycycline to induce the expression of RhoA FRET reporter. Cells were serum-starved overnight and treated with 50 ng/ml HGF for 3 hours in the presence of control DMSO-containing media. Images of different channels were acquired and data were analyzed using MetaMorph and ImageJ. The FRET signal reflecting RhoA activity is shown.
(AVI)

S4 Movie Abl inhibitor GNF2 suppresses HGF-induced RhoA activation. MDCK-FRET cells were grown in medium without doxycycline to induce the expression of RhoA FRET reporter. Cells were serum-starved overnight and treated with 50 ng/ml HGF for 3 hours in the presence of 20μm GNF2. Images of different channels were acquired and data were analyzed using MetaMorph and ImageJ. The FRET signal reflecting RhoA activity is shown. Compare to S3 Movie.
(AVI)

## References

[1] Christensen JG, Burrows J, Salgia R. c-Met as a target for human cancer and characterization of inhibitors for therapeutic intervention. Cancer Lett. 2005;225(1):1–26. Epub 2005/06/01. 10.1016/j.canlet.2004.09.044 PubMed . 15922853

[2] Birchmeier C, Birchmeier W, Gherardi E, Vande Woude GF. Met, metastasis, motility and more. Nat Rev Mol Cell Biol. 2003;4(12):915–25. Epub 2003/12/20. 10.1038/nrm1261 PubMed . 14685170

[3] Liu X, Newton RC, Scherle PA. Developing c-MET pathway inhibitors for cancer therapy: progress and challenges. Trends Mol Med. 2010;16(1):37–45. Epub 2009/12/25. 10.1016/j.molmed.2009.11.005 PubMed . 20031486

[4] Engelman JA, Zejnullahu K, Mitsudomi T, Song Y, Hyland C, Park JO, et al MET amplification leads to gefitinib resistance in lung cancer by activating ERBB3 signaling. Science. 2007;316(5827):1039–43. Epub 2007/04/28. 10.1126/science.1141478 PubMed . 17463250

[5] Comoglio PM, Giordano S, Trusolino L. Drug development of MET inhibitors: targeting oncogene addiction and expedience. Nat Rev Drug Discov. 2008;7(6):504–16. Epub 2008/05/31. 10.1038/nrd2530 PubMed . 18511928

[6] Gherardi E, Birchmeier W, Birchmeier C, Vande Woude G. Targeting MET in cancer: rationale and progress. Nat Rev Cancer. 2012;12(2):89–103. Epub 2012/01/25. 10.1038/nrc3205 PubMed . 22270953

[7] Houldsworth J, Cordon-Cardo C, Ladanyi M, Kelsen DP, Chaganti RS. Gene amplification in gastric and esophageal adenocarcinomas. Cancer Res. 1990;50(19):6417–22. Epub 1990/10/01. PubMed . 2400999

[8] Bean J, Brennan C, Shih JY, Riely G, Viale A, Wang L, et al MET amplification occurs with or without T790M mutations in EGFR mutant lung tumors with acquired resistance to gefitinib or erlotinib. Proceedings of the National Academy of Sciences of the United States of America. 2007;104(52):20932–7. Epub 2007/12/21. 10.1073/pnas.0710370104 PubMed 18093943 PMC2409244

[9] Tong CY, Hui AB, Yin XL, Pang JC, Zhu XL, Poon WS, et al Detection of oncogene amplifications in medulloblastomas by comparative genomic hybridization and array-based comparative genomic hybridization. J Neurosurg. 2004;100(2 Suppl Pediatrics):187–93. Epub 2004/02/05. 10.3171/ped.2004.100.2.0187 PubMed . 14758948

[10] Schmidt L, Duh FM, Chen F, Kishida T, Glenn G, Choyke P, et al Germline and somatic mutations in the tyrosine kinase domain of the MET proto-oncogene in papillary renal carcinomas. Nat Genet. 1997;16(1):68–73. Epub 1997/05/01. 10.1038/ng0597-68 PubMed . 9140397

[11] Park WS, Dong SM, Kim SY, Na EY, Shin MS, Pi JH, et al Somatic mutations in the kinase domain of the Met/hepatocyte growth factor receptor gene in childhood hepatocellular carcinomas. Cancer Res. 1999;59(2):307–10. Epub 1999/02/02. PubMed . 9927037

[12] Lee JH, Han SU, Cho H, Jennings B, Gerrard B, Dean M, et al A novel germ line juxtamembrane Met mutation in human gastric cancer. Oncogene. 2000;19(43):4947–53. Epub 2000/10/24. 10.1038/sj.onc.1203874 PubMed . 11042681

[13] Lai AZ, Cory S, Zhao H, Gigoux M, Monast A, Guiot MC, et al Dynamic reprogramming of signaling upon met inhibition reveals a mechanism of drug resistance in gastric cancer. Sci Signal. 2014;7(322):ra38 Epub 2014/04/24. 10.1126/scisignal.2004839 PubMed . 24757178

[14] Ueki T, Fujimoto J, Suzuki T, Yamamoto H, Okamoto E. Expression of hepatocyte growth factor and its receptor, the c-met proto-oncogene, in hepatocellular carcinoma. Hepatology. 1997;25(3):619–23. Epub 1997/03/01. 10.1002/hep.510250321 PubMed . 9049208

[15] Camp RL, Rimm EB, Rimm DL. Met expression is associated with poor outcome in patients with axillary lymph node negative breast carcinoma. Cancer. 1999;86(11):2259–65. Epub 1999/12/11. PubMed . 10590366 10.1002/(sici)1097-0142(19991201)86:11<2259::aid-cncr13>3.0.co;2-2

[16] Wielenga VJ, van der Voort R, Taher TE, Smit L, Beuling EA, van Krimpen C, et al Expression of c-Met and heparan-sulfate proteoglycan forms of CD44 in colorectal cancer. Am J Pathol. 2000;157(5):1563–73. Epub 2000/11/14. 10.1016/S0002-9440(10)64793-1 PubMed 11073815 PMC1885727

[17] Trusolino L, Bertotti A, Comoglio PM. MET signalling: principles and functions in development, organ regeneration and cancer. Nat Rev Mol Cell Biol. 2010;11(12):834–48. Epub 2010/11/26. 10.1038/nrm3012 PubMed . 21102609

[18] Rajadurai CV, Havrylov S, Zaoui K, Vaillancourt R, Stuible M, Naujokas M, et al Met receptor tyrosine kinase signals through a cortactin-Gab1 scaffold complex, to mediate invadopodia. J Cell Sci. 2012;125(Pt 12):2940–53. Epub 2012/03/01. 10.1242/jcs.100834. PubMed 22366451; PubMed Central PMCID: PMC3434810. 10.1242/jcs.100834 PMC3434810 22366451

[19] Gelsomino F, Facchinetti F, Haspinger ER, Garassino MC, Trusolino L, De Braud F, et al Targeting the MET gene for the treatment of non-small-cell lung cancer. Crit Rev Oncol Hematol. 2014;89(2):284–99. Epub 2013/12/21. 10.1016/j.critrevonc.2013.11.006. PubMed 24355409. 10.1016/j.critrevonc.2013.11.006 24355409

[20] Graveel CR, Tolbert D, Vande Woude GF. MET: a critical player in tumorigenesis and therapeutic target. 2013;5(7). Epub 2013/07/03. 10.1101/cshperspect.a009209 PubMed . PMC3685898 23818496

[21] Li R, Pendergast AM. Arg Kinase Regulates Epithelial Cell Polarity by Targeting beta1-Integrin and Small GTPase Pathways. Curr Biol. 2011;21(18):1534–42. Epub 2011/09/13. 10.1016/j.cub.2011.08.023 PubMed 21906945 PMC3189484

[22] Gu JJ, Lavau CP, Pugacheva E, Soderblom EJ, Moseley MA, Pendergast AM. Abl family kinases modulate T cell-mediated inflammation and chemokine-induced migration through the adaptor HEF1 and the GTPase Rap1. Sci Signal. 2012;5(233):ra51 Epub 2012/07/20. 10.1126/scisignal.2002632 PubMed 22810897 PMC3602906

[23] Smith-Pearson PS, Greuber EK, Yogalingam G, Pendergast AM. Abl kinases are required for invadopodia formation and chemokine-induced invasion. The Journal of biological chemistry. 2010;285(51):40201–11. Epub 2010/10/13. 10.1074/jbc.M110.147330 PubMed 20937825 PMC3001002

[24] Greuber EK, Smith-Pearson P, Wang J, Pendergast AM. Role of ABL family kinases in cancer: from leukaemia to solid tumours. Nat Rev Cancer. 2013;13(8):559–71. Epub 2013/07/12. 10.1038/nrc3563 PubMed 23842646 PMC3935732

[25] Bradley WD, Koleske AJ. Regulation of cell migration and morphogenesis by Abl-family kinases: emerging mechanisms and physiological contexts. J Cell Sci. 2009;122(Pt 19):3441–54. Epub 2009/09/18. 10.1242/jcs.039859 PubMed 19759284 PMC2746129

[26] Colicelli J. ABL tyrosine kinases: evolution of function, regulation, and specificity. Sci Signal. 2010;3(139):re6 Epub 2010/09/16. 10.1126/scisignal.3139re6 PubMed 20841568 PMC2954126

[27] Sourbier C, Ricketts CJ, Matsumoto S, Crooks DR, Liao PJ, Mannes PZ, et al Targeting ABL1-Mediated Oxidative Stress Adaptation in Fumarate Hydratase-Deficient Cancer. Cancer Cell. 2014;26(6):840–50. Epub 2014/12/10. 10.1016/j.ccell.2014.10.005 PubMed . 25490448 PMC4386283

[28] Srinivasan D, Sims JT, Plattner R. Aggressive breast cancer cells are dependent on activated Abl kinases for proliferation, anchorage-independent growth and survival. Oncogene. 2008;27(8):1095–105. Epub 2007/08/19. 10.1038/sj.onc.1210714 PubMed . 17700528

[29] Ganguly SS, Fiore LS, Sims JT, Friend JW, Srinivasan D, Thacker MA, et al c-Abl and Arg are activated in human primary melanomas, promote melanoma cell invasion via distinct pathways, and drive metastatic progression. Oncogene. 2012;31(14):1804–16. Epub 2011/09/06. 10.1038/onc.2011.361 PubMed 21892207 PMC3235241

[30] Zandy NL, Playford M, Pendergast AM. Abl tyrosine kinases regulate cell-cell adhesion through Rho GTPases. Proceedings of the National Academy of Sciences of the United States of America. 2007;104(45):17686–91. PubMed . 17965237 10.1073/pnas.0703077104 PMC2077043

[31] Jones SB, Lu HY, Lu Q. Abl tyrosine kinase promotes dendrogenesis by inducing actin cytoskeletal rearrangements in cooperation with Rho family small GTPases in hippocampal neurons. J Neurosci. 2004;24(39):8510–21. Epub 2004/10/01. 10.1523/JNEUROSCI.1264-04.2004 PubMed . 15456825 PMC6729913

[32] Lin YC, Yeckel MF, Koleske AJ. Abl2/Arg controls dendritic spine and dendrite arbor stability via distinct cytoskeletal control pathways. J Neurosci. 2013;33(5):1846–57. Epub 2013/02/01. 10.1523/JNEUROSCI.4284-12.2013 PubMed 23365224 PMC3711664

[33] Kerrisk ME, Koleske AJ. Arg kinase signaling in dendrite and synapse stabilization pathways: memory, cocaine sensitivity, and stress. Int J Biochem Cell Biol. 2013;45(11):2496–500. Epub 2013/08/07. 10.1016/j.biocel.2013.07.018 PubMed 23916785 PMC3797846

[34] Royal I, Lamarche-Vane N, Lamorte L, Kaibuchi K, Park M. Activation of cdc42, rac, PAK, and rho-kinase in response to hepatocyte growth factor differentially regulates epithelial cell colony spreading and dissociation. Mol Biol Cell. 2000;11(5):1709–25. Epub 2000/05/04. PubMed 10793146 10.1091/mbc.11.5.1709 PMC14878

[35] Schindler T, Bornmann W, Pellicena P, Miller WT, Clarkson B, Kuriyan J. Structural mechanism for STI-571 inhibition of abelson tyrosine kinase. Science. 2000;289(5486):1938–42. Epub 2000/09/16. PubMed . 10988075 10.1126/science.289.5486.1938

[36] Adrian FJ, Ding Q, Sim T, Velentza A, Sloan C, Liu Y, et al Allosteric inhibitors of Bcr-abl-dependent cell proliferation. Nat Chem Biol. 2006;2(2):95–102. Epub 2006/01/18. 10.1038/nchembio760 PubMed . 16415863

[37] Packer LM, Rana S, Hayward R, O'Hare T, Eide CA, Rebocho A, et al Nilotinib and MEK inhibitors induce synthetic lethality through paradoxical activation of RAF in drug-resistant chronic myeloid leukemia. Cancer Cell. 2011;20(6):715–27. Epub 2011/12/16. 10.1016/j.ccr.2011.11.004 PubMed 22169110 PMC3951999

[38] Zhang J, Adrian FJ, Jahnke W, Cowan-Jacob SW, Li AG, Iacob RE, et al Targeting Bcr-Abl by combining allosteric with ATP-binding-site inhibitors. Nature. 2010;463(7280):501–6. Epub 2010/01/15. 10.1038/nature08675 PubMed 20072125 PMC2901986

[39] Buchdunger E, Cioffi CL, Law N, Stover D, Ohno-Jones S, Druker BJ, et al Abl protein-tyrosine kinase inhibitor STI571 inhibits in vitro signal transduction mediated by c-kit and platelet-derived growth factor receptors. J Pharmacol Exp Ther. 2000;295(1):139–45. Epub 2000/09/19. PubMed . 10991971

[40] Zhu H, Naujokas MA, Fixman ED, Torossian K, Park M. Tyrosine 1356 in the carboxyl-terminal tail of the HGF/SF receptor is essential for the transduction of signals for cell motility and morphogenesis. J Biol Chem. 1994;269(47):29943–8. Epub 1994/11/25. PubMed . 7961992

[41] Plattner R, Koleske AJ, Kazlauskas A, Pendergast AM. Bidirectional signaling links the Abelson kinases to the platelet-derived growth factor receptor. Mol Cell Biol. 2004;24(6):2573–83. Epub 2004/03/03. PubMed 14993293 10.1128/MCB.24.6.2573-2583.2004 PMC355852

[42] Srinivasan D, Kaetzel DM, Plattner R. Reciprocal regulation of Abl and receptor tyrosine kinases. Cell Signal. 2009;21(7):1143–50. Epub 2009/03/12. 10.1016/j.cellsig.2009.03.003 PubMed 19275932 PMC2701649

[43] Ridley AJ, Comoglio PM, Hall A. Regulation of scatter factor/hepatocyte growth factor responses by Ras, Rac, and Rho in MDCK cells. Mol Cell Biol. 1995;15(2):1110–22. Epub 1995/02/01. PubMed 7823927 10.1128/mcb.15.2.1110 PMC232019

[44] de Rooij J, Kerstens A, Danuser G, Schwartz MA, Waterman-Storer CM. Integrin-dependent actomyosin contraction regulates epithelial cell scattering. The Journal of cell biology. 2005;171(1):153–64. Epub 2005/10/12. 10.1083/jcb.200506152 PubMed 16216928 PMC2171213

[45] Wells CM, Ahmed T, Masters JR, Jones GE. Rho family GTPases are activated during HGF-stimulated prostate cancer-cell scattering. Cell Motil Cytoskeleton. 2005;62(3):180–94. Epub 2005/10/08. 10.1002/cm.20095 PubMed . 16211585

[46] Wu D, Asiedu M, Wei Q. Myosin-interacting guanine exchange factor (MyoGEF) regulates the invasion activity of MDA-MB-231 breast cancer cells through activation of RhoA and RhoC. Oncogene. 2009;28(22):2219–30. Epub 2009/05/08. 10.1038/onc.2009.96 PubMed 19421144 PMC2692373

[47] Pertz O, Hodgson L, Klemke RL, Hahn KM. Spatiotemporal dynamics of RhoA activity in migrating cells. Nature. 2006;440(7087):1069–72. Epub 2006/03/21. 10.1038/nature04665 PubMed . 16547516

[48] Debnath J, Brugge JS. Modelling glandular epithelial cancers in three-dimensional cultures. Nat Rev Cancer. 2005;5(9):675–88. Epub 2005/09/09. 10.1038/nrc1695 PubMed . 16148884

[49] Pollack AL, Runyan RB, Mostov KE. Morphogenetic mechanisms of epithelial tubulogenesis: MDCK cell polarity is transiently rearranged without loss of cell-cell contact during scatter factor/hepatocyte growth factor-induced tubulogenesis. Dev Biol. 1998;204(1):64–79. Epub 1998/12/16. 10.1006/dbio.1998.9091 PubMed . 9851843

[50] Gastaldi S, Comoglio PM, Trusolino L. The Met oncogene and basal-like breast cancer: another culprit to watch out for? Breast Cancer Res. 2010;12(4):208 Epub 2010/09/02. 10.1186/bcr2617 PubMed 20804567 PMC2949647

[51] Ponzo MG, Lesurf R, Petkiewicz S, O'Malley FP, Pinnaduwage D, Andrulis IL, et al Met induces mammary tumors with diverse histologies and is associated with poor outcome and human basal breast cancer. Proceedings of the National Academy of Sciences of the United States of America. 2009;106(31):12903–8. Epub 2009/07/21. 10.1073/pnas.0810402106 PubMed 19617568 PMC2722321

[52] Knight JF, Lesurf R, Zhao H, Pinnaduwage D, Davis RR, Saleh SM, et al Met synergizes with p53 loss to induce mammary tumors that possess features of claudin-low breast cancer. Proceedings of the National Academy of Sciences of the United States of America. 2013;110(14):E1301–10. Epub 2013/03/20. 10.1073/pnas.1210353110 PubMed 23509284 PMC3619286

[53] Beaty BT, Sharma VP, Bravo-Cordero JJ, Simpson MA, Eddy RJ, Koleske AJ, et al beta1 integrin regulates Arg to promote invadopodial maturation and matrix degradation. Mol Biol Cell. 2013;24(11):1661–75, S1-11. Epub 2013/04/05. 10.1091/mbc.E12-12-0908 PubMed 23552693 PMC3667720

[54] Bell ES, Park M. Models of crk adaptor proteins in cancer. Genes Cancer. 2012;3(5–6):341–52. Epub 2012/12/12. 10.1177/1947601912459951 PubMed 23226572 PMC3513787

[55] Simpson MA, Bradley WD, Harburger D, Parsons M, Calderwood DA, Koleske AJ. Direct Interactions with the Integrin beta1 Cytoplasmic Tail Activate the Abl2/Arg Kinase. The Journal of biological chemistry. 2015. Epub 2015/02/20. 10.1074/jbc.M115.638874 PubMed . 25694433 PMC4375489

[56] Bryant DM, Roignot J, Datta A, Overeem AW, Kim M, Yu W, et al A molecular switch for the orientation of epithelial cell polarization. Dev Cell. 2014;31(2):171–87. Epub 2014/10/14. 10.1016/j.devcel.2014.08.027 PubMed 25307480 PMC4248238

[57] Kadono Y, Shibahara K, Namiki M, Watanabe Y, Seiki M, Sato H. Membrane type 1-matrix metalloproteinase is involved in the formation of hepatocyte growth factor/scatter factor-induced branching tubules in madin-darby canine kidney epithelial cells. Biochem Biophys Res Commun. 1998;251(3):681–7. Epub 1998/10/29. 10.1006/bbrc.1998.9531 PubMed . 9790969

[58] Bradley WD, Hernandez SE, Settleman J, Koleske AJ. Integrin signaling through Arg activates p190RhoGAP by promoting its binding to p120RasGAP and recruitment to the membrane. Mol Biol Cell. 2006;17(11):4827–36. Epub 2006/09/15. 10.1091/mbc.E06-02-0132 PubMed 16971514 PMC1635390

[59] Hernandez SE, Settleman J, Koleske AJ. Adhesion-dependent regulation of p190RhoGAP in the developing brain by the Abl-related gene tyrosine kinase. Curr Biol. 2004;14(8):691–6. Epub 2004/04/16. 10.1016/j.cub.2004.03.062 PubMed . 15084284

[60] Sfakianos MK, Eisman A, Gourley SL, Bradley WD, Scheetz AJ, Settleman J, et al Inhibition of Rho via Arg and p190RhoGAP in the postnatal mouse hippocampus regulates dendritic spine maturation, synapse and dendrite stability, and behavior. J Neurosci. 2007;27(41):10982–92. Epub 2007/10/12. 10.1523/JNEUROSCI.0793-07.2007 PubMed . 17928439 PMC6672862

[61] Frasca F, Vigneri P, Vella V, Vigneri R, Wang JY. Tyrosine kinase inhibitor STI571 enhances thyroid cancer cell motile response to Hepatocyte Growth Factor. Oncogene. 2001;20(29):3845–56. Epub 2001/07/06. 10.1038/sj.onc.1204531 PubMed . 11439348

[62] Cipres A, Abassi YA, Vuori K. Abl functions as a negative regulator of Met-induced cell motility via phosphorylation of the adapter protein CrkII. Cell Signal. 2007;19(8):1662–70. Epub 2007/04/03. 10.1016/j.cellsig.2007.02.011 PubMed . 17399949

[63] Furlan A, Stagni V, Hussain A, Richelme S, Conti F, Prodosmo A, et al. Abl interconnects oncogenic Met and p53 core pathways in cancer cells. Cell Death Differ. 2011. Epub 2011/04/02. 10.1038/cdd.2011.23 PubMed . 21455220 PMC3172114

[64] Zhao H, Chen MS, Lo YH, Waltz SE, Wang J, Ho PC, et al The Ron receptor tyrosine kinase activates c-Abl to promote cell proliferation through tyrosine phosphorylation of PCNA in breast cancer. Oncogene. 2014;33(11):1429–37. Epub 2013/04/02. 10.1038/onc.2013.84 PubMed 23542172 PMC4064789

[65] Tanos B, Pendergast AM. Abl tyrosine kinase regulates endocytosis of the epidermal growth factor receptor. J Biol Chem. 2006;281(43):32714–23. PubMed . 16943190 10.1074/jbc.M603126200

[66] Chislock EM, Ring C, Pendergast AM. Abl kinases are required for vascular function, Tie2 expression, and angiopoietin-1-mediated survival. Proceedings of the National Academy of Sciences of the United States of America. 2013;110(30):12432–7. Epub 2013/07/11. 10.1073/pnas.1304188110 PubMed 23840065 PMC3725093

[67] Emaduddin M, Bicknell DC, Bodmer WF, Feller SM. Cell growth, global phosphotyrosine elevation, and c-Met phosphorylation through Src family kinases in colorectal cancer cells. Proceedings of the National Academy of Sciences of the United States of America. 2008;105(7):2358–62. Epub 2008/02/09. 10.1073/pnas.0712176105 PubMed 18258742 PMC2268141

[68] Yamamoto N, Mammadova G, Song RX, Fukami Y, Sato K. Tyrosine phosphorylation of p145met mediated by EGFR and Src is required for serum-independent survival of human bladder carcinoma cells. Journal of cell science. 2006;119(Pt 22):4623–33. Epub 2006/10/26. 10.1242/jcs.03236 PubMed . 17062641

[69] Dulak AM, Gubish CT, Stabile LP, Henry C, Siegfried JM. HGF-independent potentiation of EGFR action by c-Met. Oncogene. 2011;30(33):3625–35. Epub 2011/03/23. 10.1038/onc.2011.84 PubMed 21423210 PMC3126872

[70] Plattner R, Kadlec L, DeMali KA, Kazlauskas A, Pendergast AM. c-Abl is activated by growth factors and Src family kinases and has a role in the cellular response to PDGF. Genes Dev. 1999;13(18):2400–11. Epub 1999/09/29. PubMed 10500097 10.1101/gad.13.18.2400 PMC317022

[71] Tanis KQ, Veach D, Duewel HS, Bornmann WG, Koleske AJ. Two distinct phosphorylation pathways have additive effects on Abl family kinase activation. Mol Cell Biol. 2003;23(11):3884–96. Epub 2003/05/16. PubMed 12748290 10.1128/MCB.23.11.3884-3896.2003 PMC155218

[72] Weidner KM, Di Cesare S, Sachs M, Brinkmann V, Behrens J, Birchmeier W. Interaction between Gab1 and the c-Met receptor tyrosine kinase is responsible for epithelial morphogenesis. Nature. 1996;384(6605):173–6. Epub 1996/11/14. 10.1038/384173a0 PubMed . 8906793

[73] Holohan C, Van Schaeybroeck S, Longley DB, Johnston PG. Cancer drug resistance: an evolving paradigm. Nat Rev Cancer. 2013;13(10):714–26. Epub 2013/09/26. 10.1038/nrc3599 PubMed . 24060863

[74] Dempke WC, Heinemann V. Resistance to EGF-R (erbB-1) and VEGF-R modulating agents. Eur J Cancer. 2009;45(7):1117–28. Epub 2009/01/07. 10.1016/j.ejca.2008.11.038 PubMed . 19124237

[75] Wilson TR, Fridlyand J, Yan Y, Penuel E, Burton L, Chan E, et al Widespread potential for growth-factor-driven resistance to anticancer kinase inhibitors. Nature. 2012;487(7408):505–9. Epub 2012/07/06. 10.1038/nature11249 PubMed 22763448 PMC3724525

[76] Duncan JS, Whittle MC, Nakamura K, Abell AN, Midland AA, Zawistowski JS, et al Dynamic reprogramming of the kinome in response to targeted MEK inhibition in triple-negative breast cancer. Cell. 2012;149(2):307–21. Epub 2012/04/17. 10.1016/j.cell.2012.02.053 PubMed 22500798 PMC3328787

[77] Shattuck DL, Miller JK, Carraway KL, 3rd, Sweeney C. Met receptor contributes to trastuzumab resistance of Her2-overexpressing breast cancer cells. Cancer Res. 2008;68(5):1471–7. Epub 2008/03/05. 10.1158/0008-5472.CAN-07-5962 PubMed . 18316611

[78] Mueller KL, Hunter LA, Ethier SP, Boerner JL. Met and c-Src cooperate to compensate for loss of epidermal growth factor receptor kinase activity in breast cancer cells. Cancer Res. 2008;68(9):3314–22. Epub 2008/05/03. 10.1158/0008-5472.CAN-08-0132 PubMed . 18451158 PMC3878202

[79] McDermott U, Pusapati RV, Christensen JG, Gray NS, Settleman J. Acquired resistance of non-small cell lung cancer cells to MET kinase inhibition is mediated by a switch to epidermal growth factor receptor dependency. Cancer Res. 2010;70(4):1625–34. Epub 2010/02/04. 10.1158/0008-5472.CAN-09-3620 PubMed 20124471 PMC3057521

[80] Rae JM, Creighton CJ, Meck JM, Haddad BR, Johnson MD. MDA-MB-435 cells are derived from M14 melanoma cells—a loss for breast cancer, but a boon for melanoma research. Breast Cancer Res Treat. 2007;104(1):13–9. Epub 2006/09/28. 10.1007/s10549-006-9392-8 PubMed . 17004106

[81] McLaughlin J, Cheng D, Singer O, Lukacs RU, Radu CG, Verma IM, et al Sustained suppression of Bcr-Abl-driven lymphoid leukemia by microRNA mimics. Proceedings of the National Academy of Sciences of the United States of America. 2007;104(51):20501–6. PubMed . 18079287 10.1073/pnas.0710532105 PMC2154460

[82] Smith-Pearson PS, Greuber EK, Yogalingam G, Pendergast AM. Abl kinases are required for invadopodia formation and chemokine-induced invasion. The Journal of biological chemistry. 285(51):40201–11. PubMed 10.1074/jbc.M110.147330 20937825 PMC3001002

[83] Yogalingam G, Pendergast AM. Abl kinases regulate autophagy by promoting the trafficking and function of lysosomal components. The Journal of biological chemistry. 2008;283(51):35941–53. PubMed 10.1074/jbc.M804543200 18945674 PMC2602914

[84] O'Brien LE, Yu W, Tang K, Jou TS, Zegers MM, Mostov KE. Morphological and biochemical analysis of Rac1 in three-dimensional epithelial cell cultures. Methods in enzymology. 2006;406:676–91. Epub 2006/02/14. 10.1016/S0076-6879(06)06053-8 PubMed . 16472697

[85] Hodgson L, Nalbant P, Shen F, Hahn K. Imaging and photobleach correction of Mero-CBD, sensor of endogenous Cdc42 activation. Methods in enzymology. 2006;406:140–56. PubMed . 16472656 10.1016/S0076-6879(06)06012-5

