# Supplementary figures and images for: Abl Kinases Regulate HGF/Met Signaling Required for Epithelial Cell Scattering, Tubulogenesis and Motility
Source: PLoS One. 2015 May 6;10(5):e0124960. doi: 10.1371/journal.pone.0124960 (PMC4422589; doi:10.1371/journal.pone.0124960)

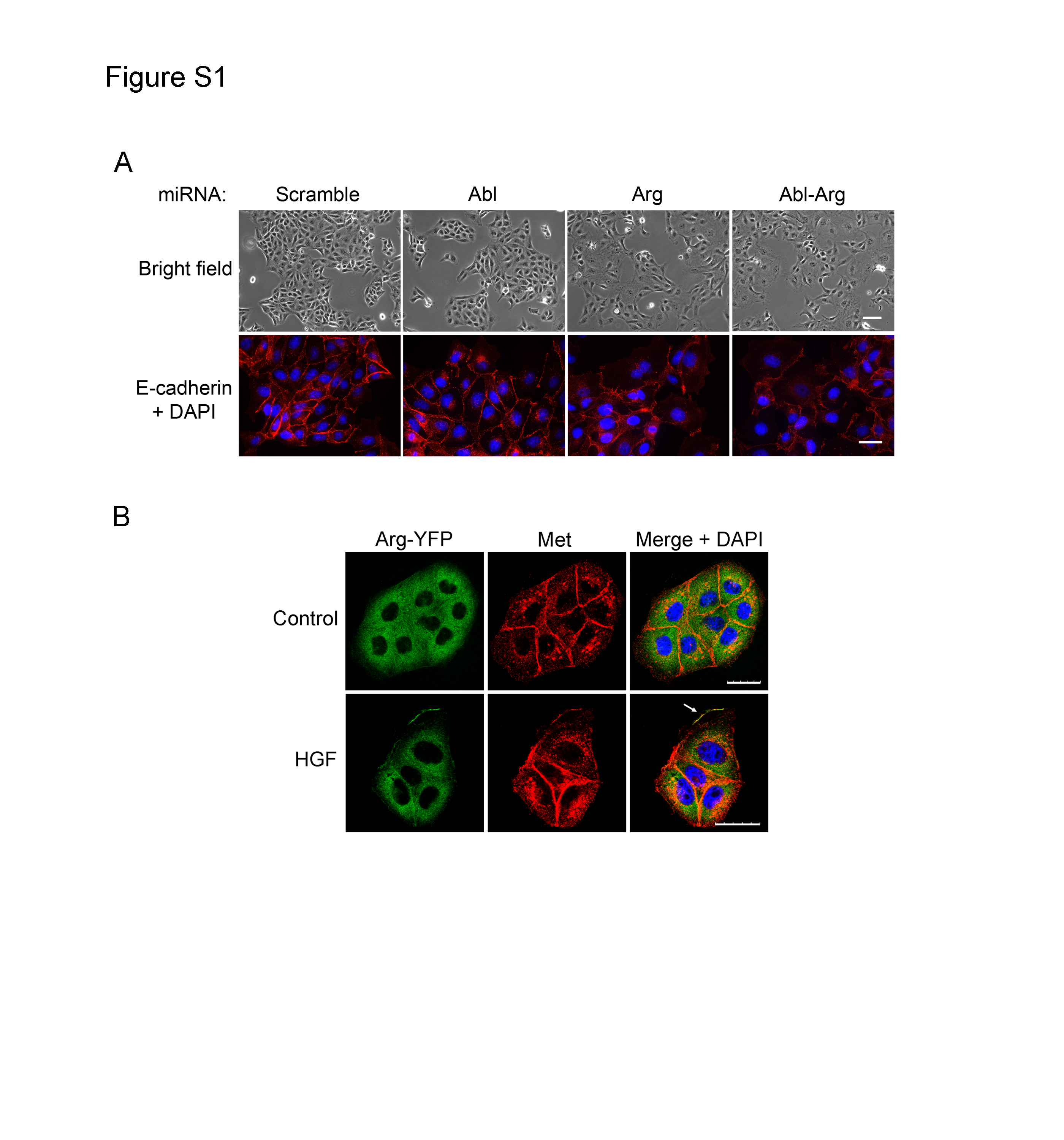

Supplement: S1 Fig — (A) MDCK cells expressing the indicated miRNAs were fixed. Bright field pictures were taken and shown in upper panels. Scale bar, 50μm. Adherens junctions were detected by staining cells with anti-E-cadherin antibody (lower panels). Scale bar, 20μm. (B) MDCK cells expressing low levels of wild-type Arg-YFP were serum-starved overnight and treated with HGF (20 ng/ml) for 10 minutes. Cells were fixed and stained for YFP and Met and visualized by confocal microscopy. Scale bars, 25μm. (TIF) [file pone.0124960.s001.tif]

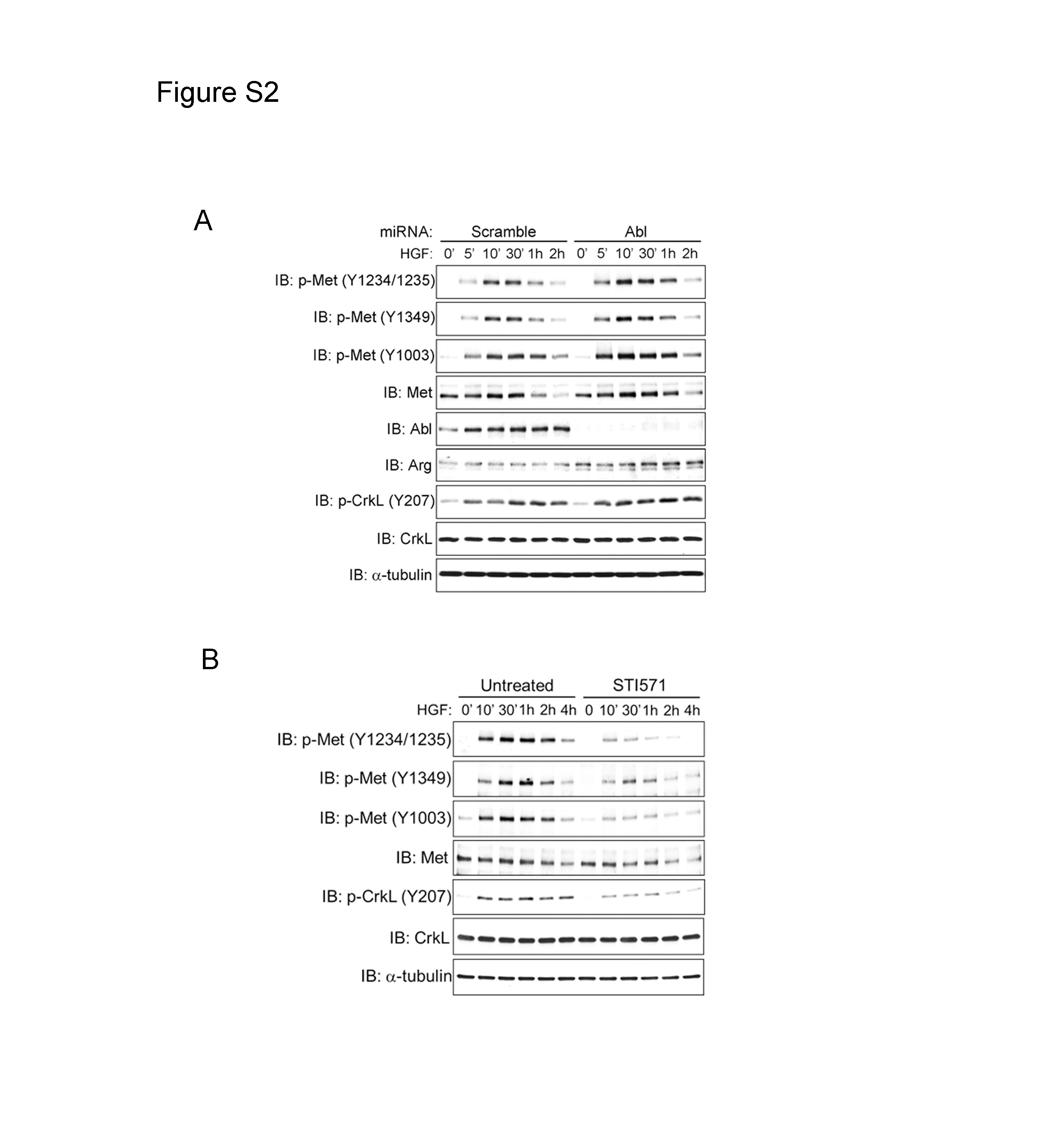

Supplement: S2 Fig — (A) Inactivation of Abl kinase alone does not affect HGF-induced Met receptor phosphorylation. Serum-starved MDCK cells expressing either scramble control or Abl miRNA were treated with 20 ng/ml HGF for the indicated times. Cells were lysed and the lysates were subjected to western blotting with the indicated antibodies. (B) Pharmacological Inhibition of Abl kinases decreases Met receptor phosphorylation following HGF stimulation. MDCK cells were serum starved with 0.25% FBS for 20h prior to stimulation with 20ng/ml HGF in the presence or absence of 15uM STI571. Cell lysates were subjected to western blotting for the indicated antibodies. Treatment with 15uM STI571 decreased tyrosine phosphorylation of Y207 of CrkL as well as Y1349, 1003 and 1234/1235 residues of the c-Met receptor. (TIF) [file pone.0124960.s002.tif]

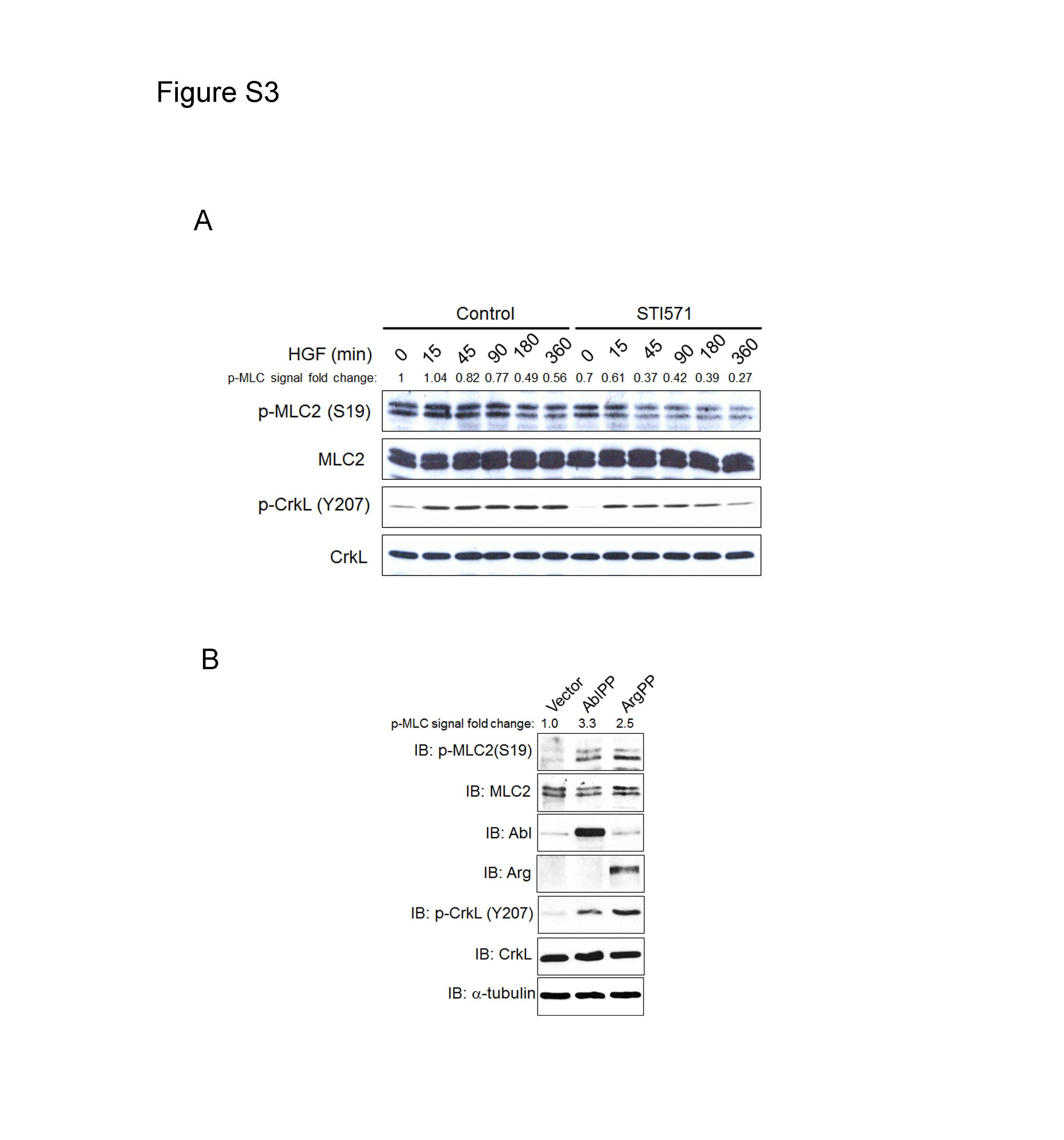

Supplement: S3 Fig — (A) Inhibition of Abl kinases decreased myosin light chain (MLC) phosphorylation in MDCK cells upon HGF treatment. Serum starved MDCK cells were treated with HGF (20ng/ml) in the presence or absence of 10uM STI571. Cell lysates were subjected to western blotting for the indicated antibodies. (B) Active mutants of Abl/Arg kinases induced hyperphosphorylation of the myosin light chain. MDCK cells expressing either vector control, or constitutively active Abl-PP or Arg-PP were lysed and the lysates were subjected to western blotting with the indicated antibodies. (TIF) [file pone.0124960.s003.tif]

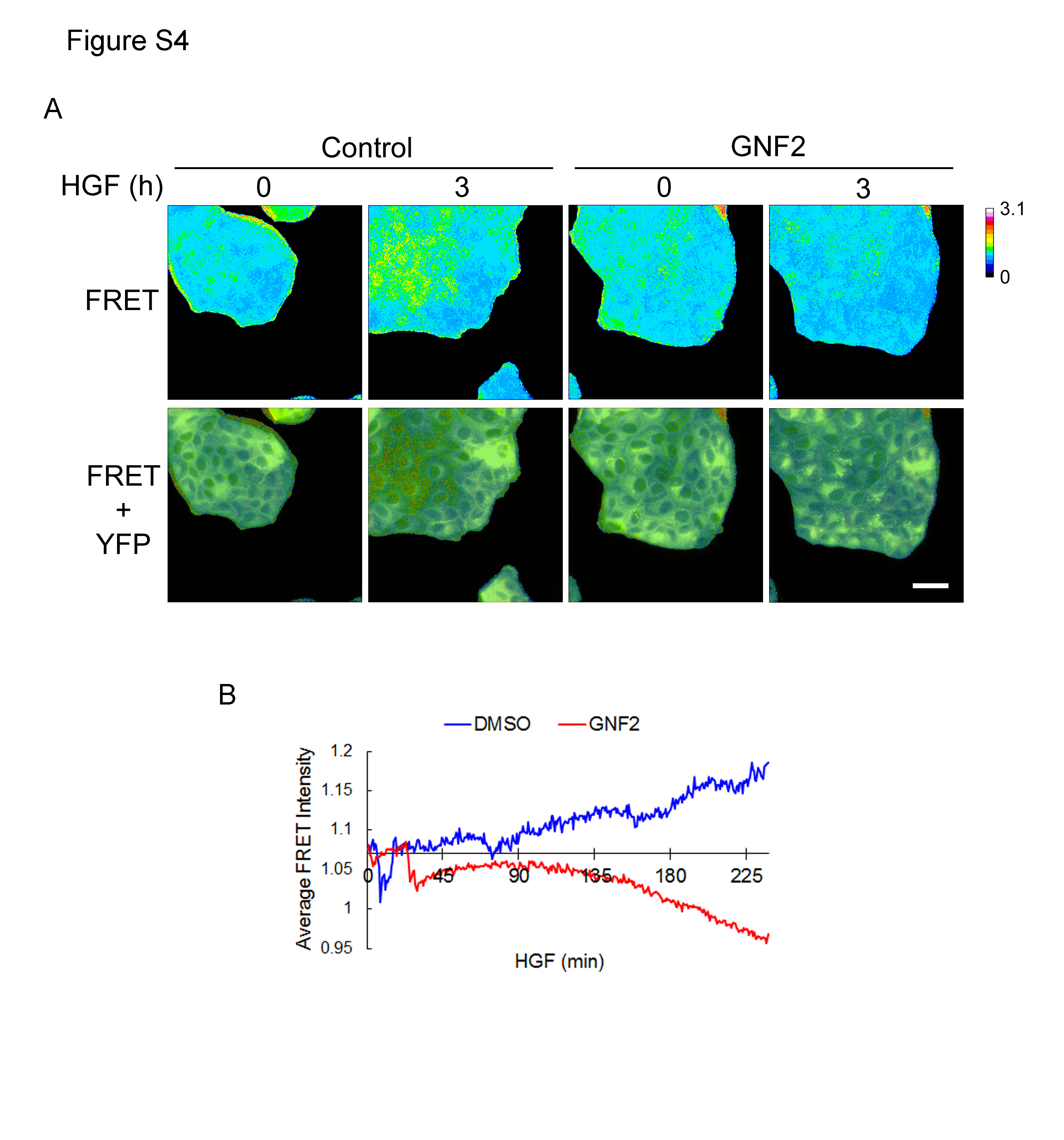

Supplement: S4 Fig — (A) MDCK-FRET cells were grown in medium without doxycycline to induce the expression of RhoA FRET reporter. Cells were serum-starved overnight and treated with HGF (50 ng/ml) for 3 hours in presence or absence of 20μm GNF2. Images of different channels were acquired and data were analyzed using MetaMorph software. The FRET signal reflecting RhoA activity is shown. YFP signal is used to define cell bodies. Scale bar, 15μm. (B) quantification of the FRET signal over time from each experimental group in (A) is shown. (TIF) [file pone.0124960.s004.tif]

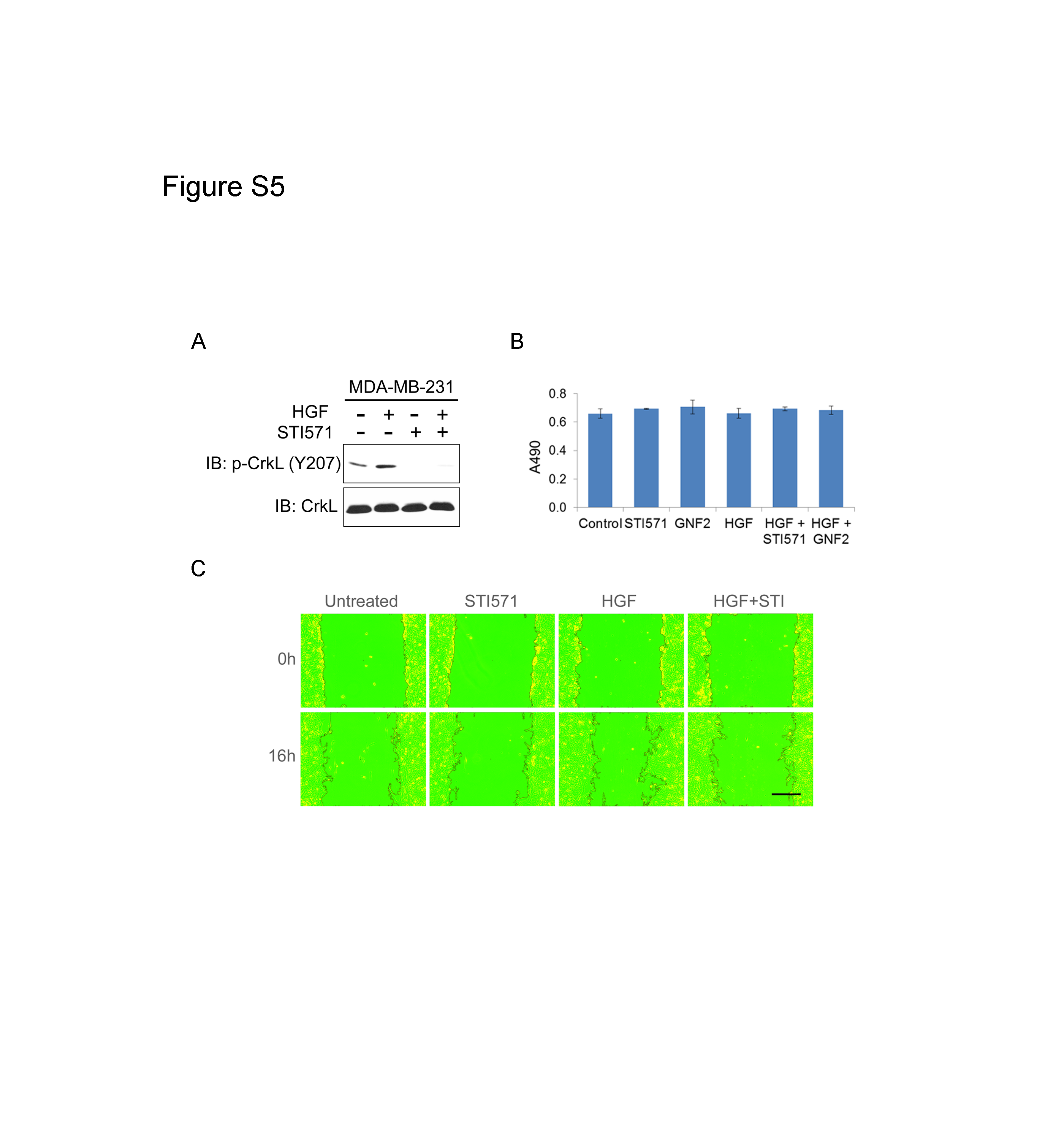

Supplement: S5 Fig — (A) Abl kinases are activated by Met in MDA-MB-231 cells. Serum-starved MDA-MB-231 cells were treated with HGF for 30 min with or without 10μM STI571. Cell lysates were subjected to western blotting with the indicated antibodies. (B) MDA-MB-231 cells (5,000) were plated in each well of a 96-well plate and were left either untreated or treated with HGF, with or without Abl kinase inhibitors. After 24 hours, cells were subjected to the MTS cell viability assay, and A490 values were measured and analyzed by one-way ANOVA. Error bars represent mean ± S.D. (C) A wound was generated within a confluent monolayer of serum-starved MDA-MB-231 cells. Indicated cells were pre-treated with STI571 and then allowed to migrate for 16 hours as indicated. Bright field pictures were acquired and the images were analyzed with ImageJ. Scale bar, 200μm. (TIF) [file pone.0124960.s005.tif]

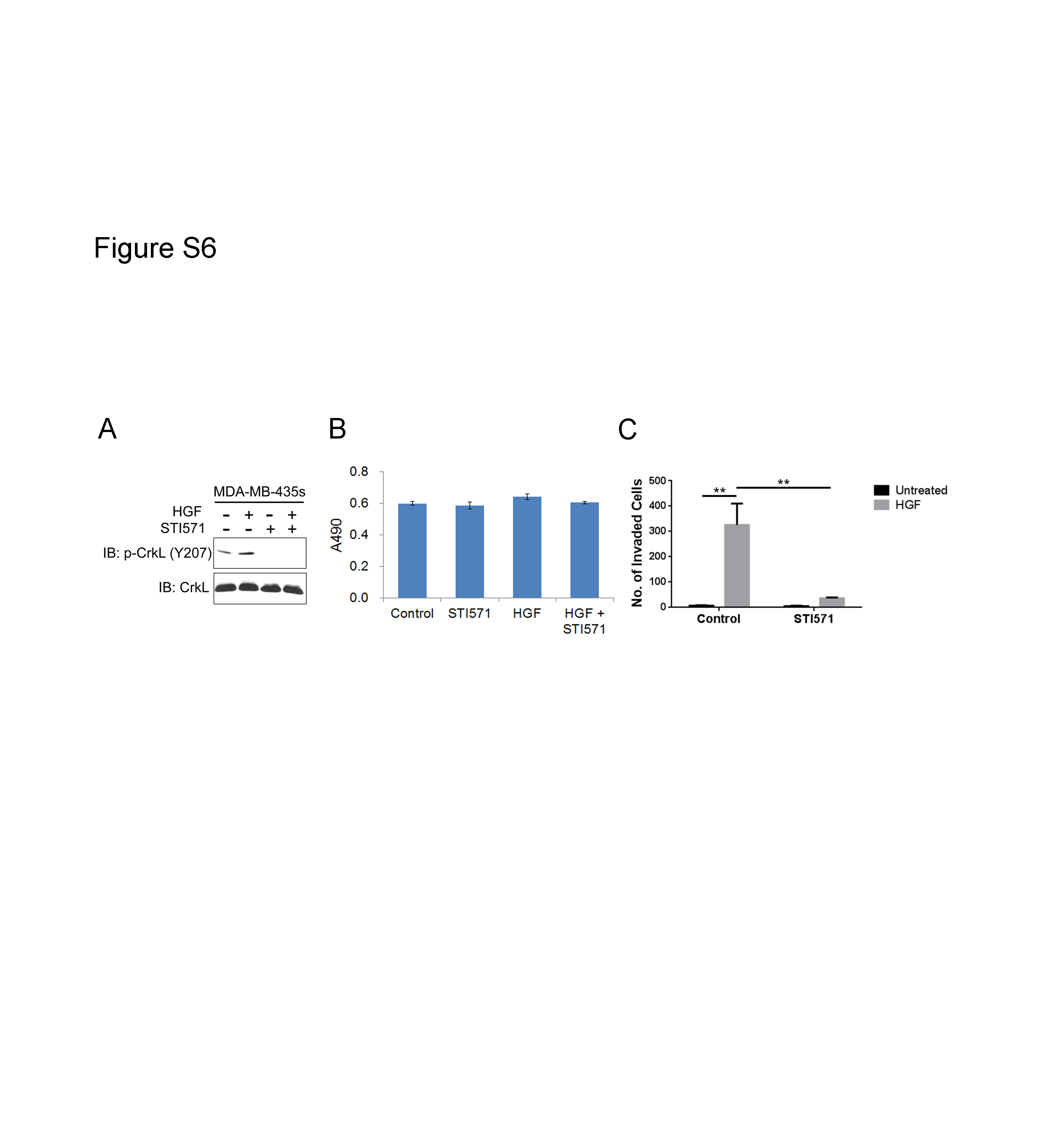

Supplement: S6 Fig — (A) Serum-starved MDA-MB-435s cells were treated with HGF for 30 min with or without 10μM STI571. Cell lysates were subjected to western blotting with the indicated antibodies. (B) MDA-MB-435s cells (5,000) were plated in each well of a 96-well plate and left either untreated or treated with HGF with or without STI571. After 24 hours, cells were subjected to the MTS cell viability assay and A490 values were measured and analyzed by one-way ANOVA. Error bars represent mean ± S.D. (C) Serum-starved MDA-MB-435s cells were plated in the upper well of the matrigel invasion chambers in the presence or absence of STI571. HGF was added in the lower chambers with or without STI571, and after 48 hours, cells invading the undersurface were quantified and analyzed by two-way ANOVA followed by Bonferroni post-test. **P<0.01. Error bars represent mean (n = 3) ± S.E.M. (TIF) [file pone.0124960.s006.tif]
